# Enzyme Activities at Different Stages of Plant Biomass Decomposition in Three Species of Fungus-Growing Termites

**DOI:** 10.1128/AEM.01815-17

**Published:** 2018-02-14

**Authors:** Rafael R. da Costa, Haofu Hu, Bo Pilgaard, Sabine M. E. Vreeburg, Julia Schückel, Kristine S. K. Pedersen, Stjepan K. Kračun, Peter K. Busk, Jesper Harholt, Panagiotis Sapountzis, Lene Lange, Duur K. Aanen, Michael Poulsen

**Affiliations:** a Centre for Social Evolution, Section for Ecology and Evolution, Department of Biology, University of Copenhagen, Copenhagen, Denmark; b Department of Chemical and Biochemical Engineering, Technical University of Denmark, Kongens Lyngby, Denmark; c Laboratory of Genetics, Wageningen University, Wageningen, The Netherlands; d Department of Plant and Environmental Sciences, Section for Plant Glycobiology, University of Copenhagen, Frederiksberg, Denmark; e Carlsberg Research Laboratory, Copenhagen, Denmark; University of Georgia

**Keywords:** AZCL, chromogenic substrates, HPLC, Macrotermes, Odontotermes, peptide pattern recognition, plant substrate, RNA-seq, symbiosis, Termitomyces

## Abstract

Fungus-growing termites rely on mutualistic fungi of the genus Termitomyces and gut microbes for plant biomass degradation. Due to a certain degree of symbiont complementarity, this tripartite symbiosis has evolved as a complex bioreactor, enabling decomposition of nearly any plant polymer, likely contributing to the success of the termites as one of the main plant decomposers in the Old World. In this study, we evaluated which plant polymers are decomposed and which enzymes are active during the decomposition process in two major genera of fungus-growing termites. We found a diversity of active enzymes at different stages of decomposition and a consistent decrease in plant components during the decomposition process. Furthermore, our findings are consistent with the hypothesis that termites transport enzymes from the older mature parts of the fungus comb through young worker guts to freshly inoculated plant substrate. However, preliminary fungal RNA sequencing (RNA-seq) analyses suggest that this likely transport is supplemented with enzymes produced *in situ*. Our findings support that the maintenance of an external fungus comb, inoculated with an optimal mixture of plant material, fungal spores, and enzymes, is likely the key to the extraordinarily efficient plant decomposition in fungus-growing termites.

**IMPORTANCE** Fungus-growing termites have a substantial ecological footprint in the Old World (sub)tropics due to their ability to decompose dead plant material. Through the establishment of an elaborate plant biomass inoculation strategy and through fungal and bacterial enzyme contributions, this farming symbiosis has become an efficient and versatile aerobic bioreactor for plant substrate conversion. Since little is known about what enzymes are expressed and where they are active at different stages of the decomposition process, we used enzyme assays, transcriptomics, and plant content measurements to shed light on how this decomposition of plant substrate is so effectively accomplished.

## INTRODUCTION

Plant biomass is one of the most nutritious and abundant carbon sources utilized by a range of organisms (1). Primarily consisting of cell walls, plant substrates present a complex structure of polysaccharides, proteins, and lignin, differing between plant species in their monomeric composition and linkages (2). The complex arrangements of different plant cell wall polymers make them resistant to degradation, yet many microorganisms can effectively degrade these polysaccharides through the secretion of enzymes that cleave complex saccharides to release oligo-, di-, and monosaccharides (1). Most animals do not have the necessary enzymes to break down recalcitrant plant-derived components for nutrition (3) and thus coopt microbial symbionts for plant biomass decomposition (1, 4).

Fungal cultivation in termites evolved ca. 30 million years ago in sub-Saharan Africa, when the subfamily Macrotermitinae engaged in a mutualistic association with a fungus of the genus Termitomyces (Agaricales, Lyophyllaceae) (5, 6). The termites have become major biomass decomposers in the Old World (7, 8), where they play an important role in the turnover of dead plant material (9–11). They decompose up to 90% of the available dead plant material in African savannahs (12), with a consequently major impact on carbon cycling (13, 14). All 11 fungus-growing termite genera occur in Africa, four genera occur in Asia, and approximately 330 species have been described (15, 16). Approximately 40 species of Termitomyces have been described, all of which engage in mutualistic associations with the Macrotermitinae (17). Termitomyces spp. serve as the primary plant decomposers and as the main food source for the termites (18, 19). In addition to Termitomyces, fungus-growing termites harbor a complex and codiversified gut microbiota (20–22) that complements the plant decomposition properties of Termitomyces (23, 24).

Members of the two major fungus-growing termite genera, Macrotermes and Odontotermes, process plant biomass in a similar way (25–27), involving two gut passages and external decomposition in fungal gardens (combs) (28, 29). Old workers collect plant substrate and transport this to the mound (28, 30). Within the mound, young workers ingest the plant material along with asexual Termitomyces spores produced in fungal nodules in the mature parts of the fungal comb (31). This mixture passes through young termite gut (first gut passage), which possibly contributes to lignin cleavage (32), and the resulting excrements are used to build the fungal comb (here referred to as “fresh comb”) (28). Termitomyces spp. produce plant biomass-degrading enzymes (24, 33–35) and possibly also cleave lignin as they grow during comb maturation (19, 35–38). After maturation of the fungal comb, here referred to as “old comb,” it is consumed by old workers in a second gut passage, after which essentially all organic material is utilized (28). Gut microbial enzymes are believed to facilitate final plant decomposition during this second gut passage and to contribute to fungal cell wall degradation (23, 24).

The first gut passage serves as an effective means by which the termites ensure that the plant substrate is densely inoculated with Termitomyces spores; however, this gut passage has also been proposed to contribute to the transport of carbohydrate-active enzymes from nodules to fresh comb to boost initial plant decomposition (29). This transport of enzymes could be complemented with enzymes produced by Termitomyces mycelium within the comb. Here, we used enzyme assays to investigate which enzymes are active and which plant components are broken down at different stages of the decomposition process, and we supplemented this with fungal RNA sequencing (RNA-seq) data to shed light on the locations of fungal carbohydrate-enzyme expression.

## RESULTS

### Odontotermes species identification.

In addition to two nests (Od127 and Od128) already identified as Odontotermes spp. by Otani et al. (22), we successfully amplified the cytochrome oxidase II (COII) gene from termites derived from four Odontotermes nests sampled in 2015, one nest of which was Odontotermes sp. (Od159) and three of which were Odontotermes cf. badius (Od145, Od150, and Od151), determined by their phylogenetic placement (see Fig. S1 in the supplemental material) (sequences available in GenBank with accession numbers MF092801 to MF092804). Of the termites collected at the 18 foraging sites (Table 1), five termites were Odontotermes spp. and three termites were Odontotermes cf. badius (GenBank accession numbers MF092793 to MF092800) (Fig. S1).

**TABLE 1:** Forage substrate sampling information, type of substrate, geographical location, termite species, GenBank accession numbers for Odontotermes foragers identified with cytochrome oxidase II gene, and date of substrate collection

Type of substrate	GPS coordinates^*a*^	Location	Forager organism	GenBank accession no.^*b*^	Collection date (day-mo-yr)
Dry wood	−25.729100, 28.240883	Rietondale	Odontotermes sp.	MF092793	18-01-2016
Dry wood	−25.728533, 28.240733	Rietondale	Odontotermes cf. badius	MF092794	18-01-2016
Dry wood	−25.728017, 28.240300	Rietondale	Odontotermes cf. badius	MF092795	18-01-2016
Dry wood	−25.729050, 28.242050	Rietondale	Odontotermes sp.	MF092796	18-01-2016
Dry wood	−25.729683, 28.240550	Rietondale	Odontotermes sp.	MF092797	18-01-2016
Decaying wood	−26.815483, 30.711000	Iswepe	M. natalensis	NA	24-01-2016
Cow dung	−26.815133, 30.711283	Iswepe	M. natalensis	NA	24-01-2016
Bark	−26.815117, 30.711300	Iswepe	M. natalensis	NA	24-01-2016
Bark	−26.815183, 30.711483	Iswepe	M. natalensis	NA	24-01-2016
Dry wood	−26.813933, 30.710567	Iswepe	M. natalensis	NA	24-01-2016
Cow dung	−25.741650, 28.260083	Experimental farm	Odontotermes cf. badius	MF092798	01-02-2016
Cow dung	−24.661583, 28.793167	Mookgophong	M. natalensis	NA	03-02-2016
Cow dung	−24.674383, 28.804583	Mookgophong	Odontotermes sp.	MF092799	03-02-2016
Dry wood	−25.728967, 28.235350	Rietondale	M. natalensis	NA	05-02-2016
Dry wood	−25.729733, 28.235433	Rietondale	M. natalensis	NA	05-02-2016
Decaying wood	−25.731550, 28.235600	Rietondale	M. natalensis	NA	05-02-2016
Dry wood	−25.732233, 28.235667	Rietondale	M. natalensis	NA	05-02-2016
Dry wood	−25.732550, 28.235517	Rietondale	Odontotermes sp.	MF092800	05-02-2016

a GPS, global positioning system.

b NA, not available.

### RNA sequencing and peptide pattern recognition-based Hotpep analysis.

Since we were able to sequence mRNA from only three termite nests, one colony of Macrotermes natalensis and two of an Odontotermes sp., the results of the RNA-seq remains preliminary, but we include it to supplement the findings from the enzyme and content analyses. Nodules, fresh comb, and old comb of the fungal symbiont of both M. natalensis and Odontotermes sp. showed the expression of a wide spectrum of biomass-degrading enzymes, including targeting lignin, cellulose, hemicellulose, and pectin (Tables 2 and 3; see also Tables S5 and S6 in the supplemental material). Other key carbohydrate-active enzymes (CAZymes) in the modification of the plant cell wall during the decomposition process include auxiliary activities (AAs), such as laccases, versatile peroxidase, alcohol oxidase, and lytic polysaccharide monooxygenases (LPMOs), polysaccharide lyases (PLs) (mainly pectase lyase and poly-β-d-mannuronate lyase targeting pectin), and carbohydrate esterases (CEs) (Tables S5 and S6). Transcripts of a full set of enzyme functions, targeting all plant components, were observed for all three nests (Tables 2 and 3).

**TABLE 2:** Expression level and distribution of transcripts on target substrate Termitomyces fungal samples from Macrotermes natalensis and Odontotermes species^*a*^

Classification	Expression level (transcripts per million)									No. of transcript sequences placed in different CAZyme families								
	M. natalensis Mn156			Odontotermes sp. Od127			Odontotermes sp. Od128			M. natalensis Mn156			Odontotermes sp. Od127			Odontotermes sp. Od128		
	Nod.	F.C.	O.C.	Nod.	F.C.	O.C.	Nod.	F.C.	O.C.	Nod.	F.C.	O.C.	Nod.	F.C.	O.C.	Nod.	F.C.	O.C.
Functional classification (EC no.)																		
Auxiliary activities	1,638	2,623	4,504	140.7	7,116	1,846	2,054	6,176	2,872	194	151	82	52	108	123	157	140	106
Polysaccharide lyases	738.1	1,322	1,208	21.86	1,691	261.8	394.0	130.6	183.4	49	42	28	7	24	54	23	22	21
Carbohydrate esterases	1,638	9,538	13,117	407.8	20,588	5,699	6,296	13,689	8,204	83	72	36	20	62	80	85	81	48
Glycoside hydrolases	6,705	7,880	10,510	7,439	13,675	37,016	31,214	7,483	45,298	944	825	466	294	470	649	585	449	371
Total	10,719	21,363	29,339	8,009	43,070	44,822	39,958	27,479	56,557	1,270	1,090	612	373	664	906	850	692	546
Substrate targets																		
Starch	406.9	462.5	380.2	224.0	499.0	717.2	2,864	223.5	358.7	38	37	13	25	40	27	50	28	34
Pectin	626.3	766.0	1,080	24.00	1,127	1,413	542.4	834.6	2,045	144	147	58	13	138	68	64	59	67
Arabinogalactan	469.9	351.1	713.0	31.59	1,060	675.4	158.2	379.0	930.0	94	91	42	15	92	22	44	26	20
Lignin	600.4	2,352	2,822	65.71	3,953	1,855	1,686	4,722	1,422	146	92	74	20	82	64	82	50	74
Cellulose	1,571	1,629	2,816	517	3,961	20,365	16,420	1,639	30,990	228	217	105	47	138	120	136	85	135
Hemicellulose	1,166	1,957	3,256	107.8	4,059	8,605	6,272	1,306	10,723	198	126	84	29	153	61	115	49	70
Plant-mannan	185.1	145.7	185.5	31.7	394.1	321.4	20.2	58.2	220.3	36	15	3	5	27	14	9	13	13
Total	5,026	7,664	11,253	1,001	15,054	33,952	27,963	9,162	46,689	884	725	379	154	670	376	500	310	413

a For the full results, see Tables S5 and S6. Nod., fungal nodules; F.C., fresh comb; O.C., old comb.

**TABLE 3:**
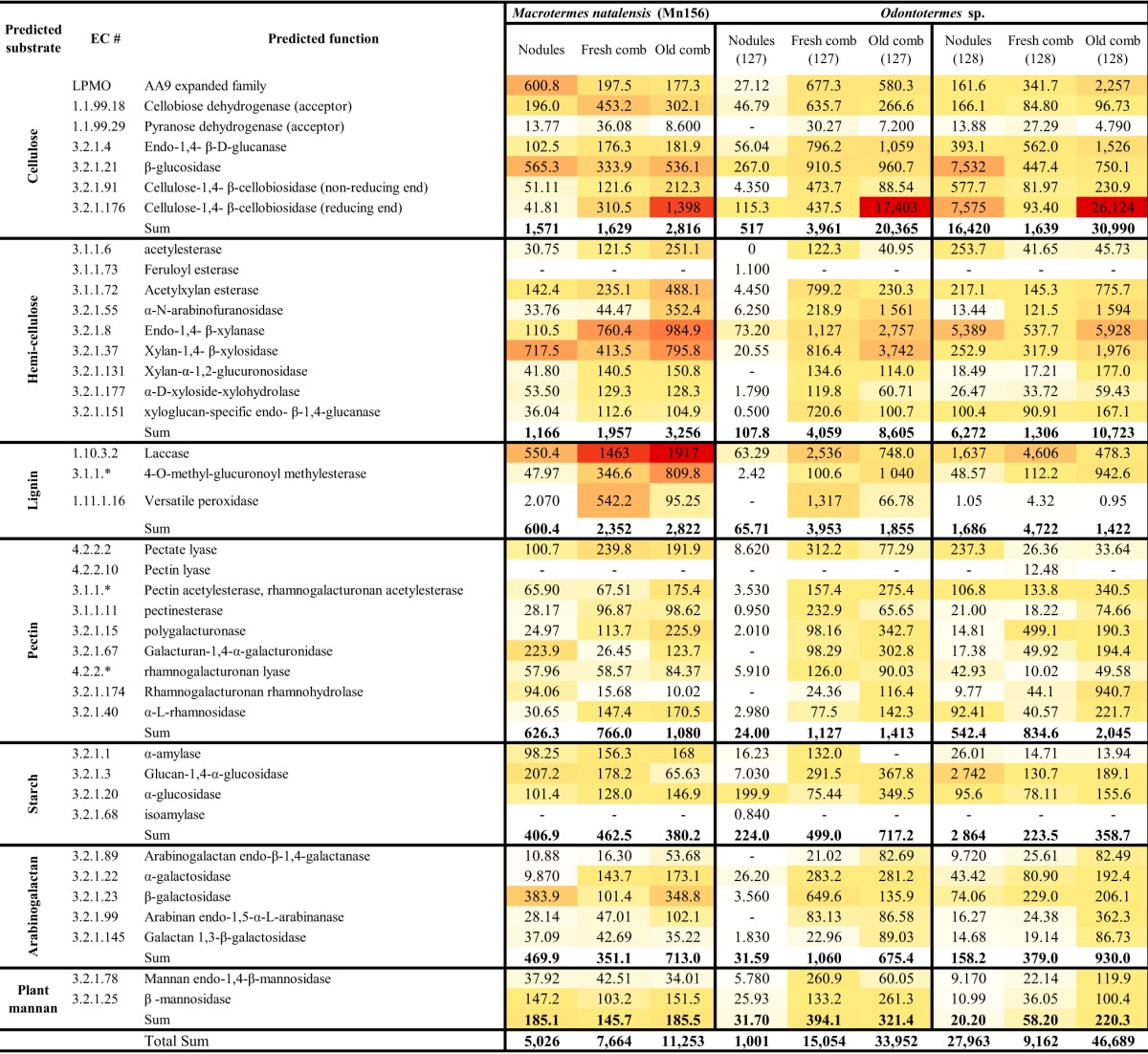
Expression levels of CAZymes across different sites of the decomposition process in one colony of Macrotermes natalensis and two Odontotermes sp. colonies^*a*^

a For the full results, see Tables S6 and S7. Nod., fungal nodules; F.C., fresh comb; O.C., old comb. White shading represents the lowest number of transcripts, yellow represents an intermediate number of transcripts, and red represents the highest number of transcripts.

Some patterns emerged from the expression analyses comparing fresh comb, nodules, and old comb: enzymes targeting cellulose, particularly cellulose 1,4-β-cellobiosidase (reducing end) (EC 3.2.1.176), were by far the most highly expressed, with highest expression in old comb, followed by a laccase (EC 1.10.3.2), which had the highest expression in fresh comb in the two Odontotermes nests but highest in M. natalensis old comb. Following these were enzymes targeting hemicellulose, which also in two out of three nests were most expressed in the old comb (Tables 2 and 3).

### Enzyme capacities across different stages in the decomposition process.

**FIG 1:**
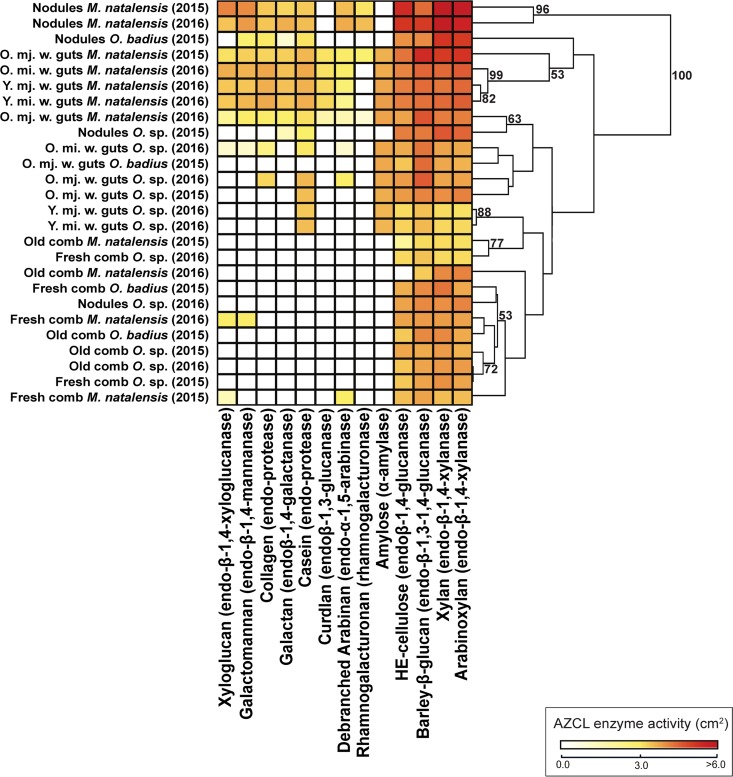
Carbohydrate-active enzyme activities through the plant decomposition process. (a) Heatmap of enzyme diversity and activity in nodules and guts from different fungus-growing termite species, and similarity analyses based on Euclidean clustering with bootstrap support after 10,000 permutations. Shown on the bottom are the AZCL substrates and the enzymes targeting them, respectively. O. (when not used as a genus abbreviation), old; Y., young; mj., major; mi., minor; w., worker.

The highest enzyme activities were identified against xylan, arabinoxylan, barley β-glucan, and hydroxyethyl cellulose (HE-cellulose), and these were highest in fungal nodules, followed by guts, fresh combs, and old combs (Fig. 1 and Table S7). The degradation activities of amylose [χ^2^_(2)_ = 3.899, *P* = 1.000], arabinoxylan [*F*_(2)_ = 2.494, *P* = 1.000], barley β-glucan [χ^2^_(2)_ = 2.710, *P* = 1.000], casein [*F*_(2)_ = 9.279, *P* = 0.7670], debranched arabinan [χ^2^_(2)_ = 8.956, *P* = 0.1476], and xylan [*F*_(2)_ = 3.124, *P* = 1.000] were not significantly different across termite species (Fig. 1 and Table S4). In contrast, enzyme activities for HE-cellulose [*F*_(2)_ = 10.95, *P* < 0.0001], collagen [χ^2^_(2)_ = 33.08, *P* < 0.0001], curdlan [χ^2^_(2)_ = 47.50, *P* < 0.0001], galactomannan [χ^2^_(2)_ = 60.51, *P* < 0.0001], galactan [χ^2^_(2)_ = 13.28, *P* < 0.0001], xyloglucan [χ^2^_(2)_ = 75.32, *P* < 0.0001], and rhamnogalacturonan [χ^2^_(2)_ = 13.28, *P* = 0.0169] were significantly different between termite species. The enzyme activities of worker guts, nodules, fresh comb, and old comb were significantly different across all azurine-cross-linked (AZCL) substrates (Table S4), except for debranched arabinan [χ^2^_(6)_ = 15.05, *P* = 0.2579].

**FIG 2:**
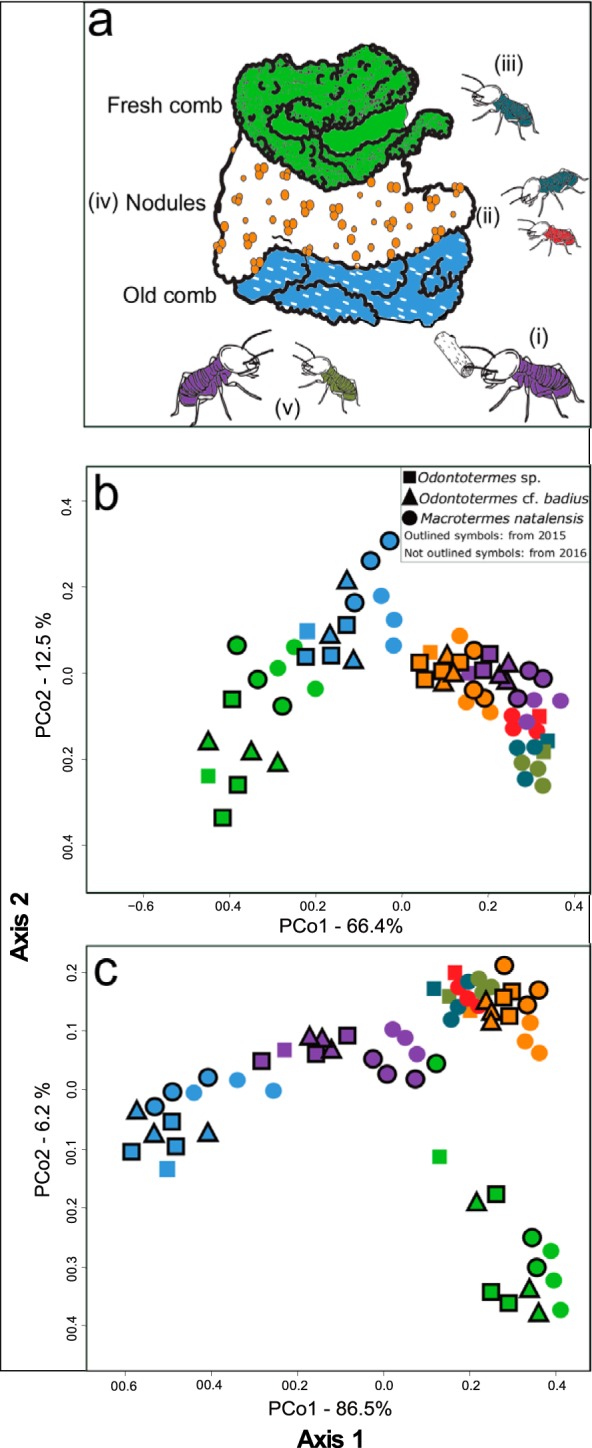
Enzyme activities across different stages of the decomposition process. (a) Schematic illustration of the decomposition process within a fungus-growing termite mound: (i) old major workers collect plant substrate from the surroundings of the mound, and (ii) this plant substrate is processed by young and old minor workers. These minor workers ingest the plant substrate along with Termitomyces asexual spores found in the nodules. (iii) This mixture passes through the termite gut (first gut passage) and is deposited as fresh comb. (iv) Once this mixture is inoculated in the fresh comb, Termitomyces spp. break down complex plant cell wall components, and, as the comb matures, new nodules are produced. (v) When the plant substrate is utilized by Termitomyces spp., old and young major (dark green) workers feed on the old part of the fungus comb (blue), and after a second gut passage, all the organic matter is essentially decomposed. (b) PCoA of AZCL enzyme activities in colony components and worker castes and ages. (c) PCoA of enzyme activities in colony components and worker castes and ages after normalization of fungus comb enzyme activity based on the relative abundance of fungus biomass. Purple, old major workers; dark green, young major worker; red, young minor worker; petrol blue, old minor workers; orange, nodules; light green, fresh comb.

Euclidean principal-coordinate analysis (PCoA) and Shannon index analyses [*F*_(6)_ = 52.86, *P* < 0.0001] showed distinct enzyme capacities between nodules, old, young, minor, and major worker guts, and fresh and old fungus comb samples associated with different stages in the decomposition process (Fig. 1 and 2a and b). Nodules, guts, fresh comb, and old comb were significantly different for almost all enzyme activities (Table S4). Enzyme diversity and activities were similar in fresh and old combs; however, the differences found (e.g., in enzyme activities to cleave debranched arabinan and galactomannan exclusively found in the fresh comb) led to separate clusters in the PCoA (Fig. 2b). Enzyme activities and diversity were similar in nodules and guts from all termite species, and they clustered together [one-way permutational multivariate analysis of variance (PERMANOVA), *F*_(7)_ *=* 3.957, *P* = 1.0000] (Fig. 1 and 2b). Worker guts clustered relatively close to each other and to nodules; however, there was a tendency toward old major worker guts separating from other worker guts (Fig. 1 and 2b). After correction of fresh-comb AZCL activities based on the ratio of glucosamine in old and fresh combs (up to almost three times higher in old comb; Table S9), comb samples remained as two distinct clusters, with old combs being more similar to old major worker guts and fresh comb being more similar to nodules and old and young minor worker guts (Fig. 2c).

Chromogenic polysaccharide hydrogel (CPH) assay results corroborated our AZCL enzyme assay results; however, galactomannan-, xyloglucan-, and rhamnogalacturonan-degrading activities were not statistically different across colony components [*F*_(6)_ = 3.227, *P* = 0.870 for galactomannan, and χ^2^_(6)_ = 9.353, *P* = 1.000 for xyloglucan, and χ^2^_(6)_ = 14.24, *P* = 1.000]. As we only had sufficient material to carry out CPH on M. natalensis (Table S8), our main analyses and discussion focus on the AZCL results. The comparisons of the results of AZCL and CPH (Fig. S2a) indicated that (i) although AZCL and CPH assays use different measurement scales (square centimeters and absorbance), comparable patterns were obtained (correlation analyses, *R*^2^ = 56.5%), and (ii) some activities were detected with the CPH assay that were absent in the AZCL assay; e.g., amylase was only detected with AZCL in guts, while the CPH assay detected activities across all colony components (Tables S7 and S8). The clustering analysis of CPH and AZCL compositions (Fig. S2b) showed that the enzyme activity estimates were largely comparable.

### Which plant polymers are decomposed in the fungus-growing termite symbiosis?

**FIG 3:**
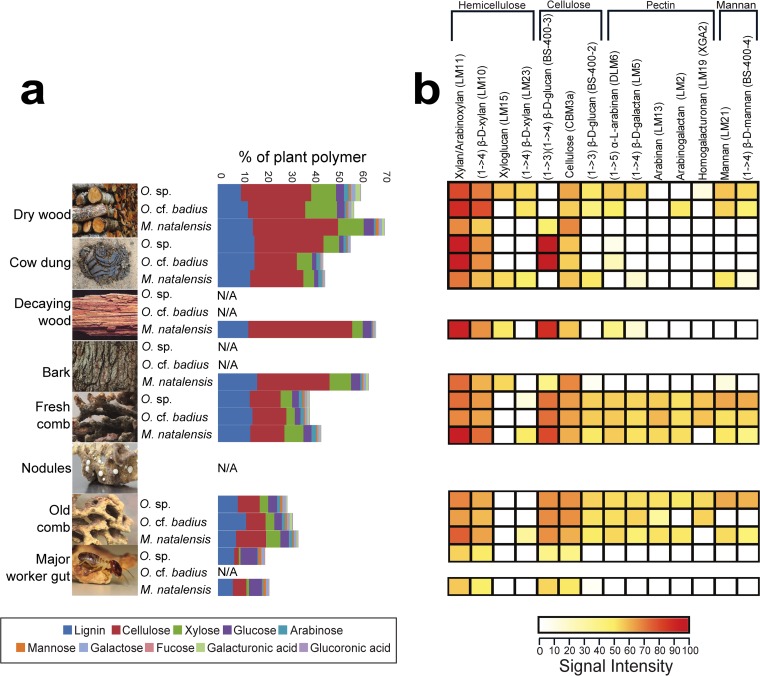
Plant biomass degradation in fungus-growing termites. (a) Content of polysaccharides expressed in % per gram of AIR sample. Cellulose content was measured by using 4% sulfuric acid hydrolysis, lignin was measured using acetyl-bromide, and noncellulosic polymers were measured using trifluoroacetic acid. Although these analyses were not performed on nodules, we include an image of them to show their presence within the comb. (b) CoMPP heatmap illustrating the distribution and relative amount of plant polymers in forage material, fungus comb, and termite guts based on NaOH extraction. The spot signal values (Table S5) are correlated to color intensity. Polysaccharide epitopes and their monoclonal antibodies are shown above. The values correspond to averages of nests (M. natalensis, *n* = 6; Odontotermes sp., *n* = 4; and Odontotermes cf. badius, *n* = 3) and forage substrate based on its type (dry wood, *n* = 4, *n* = 4, *n* = 2; and cow dung *n* = 2, *n* = 1, *n* = 1; for M. natalensis, Odontotermes sp., and Odontotermes cf. badius, respectively; decaying wood and bark, *n* = 1 for M. natalensis) (Table 4). N/A, not applicable.

We were able to characterize 40 to 64% of the plant-derived component content in foraged substrates (Fig. 3a and Table S10), with the remaining content most likely being microbial biomass, soluble sugars, and ash. Cellulose, lignin, xylose (derived from xylan), and glucose were the most abundant compounds in all forage substrate types (average ± standard deviation, 27.9% ± 2.2%, 12.2% ± 1.0%, 8.2% ± 1.1%, and 3.0% ± 0.3%) (Fig. 3a), but lignin was the only one of these major components that was not significantly different between forage types (Table S10). The minor components arabinose, mannose, galactose, fucose, glucuronic acid, and galacturonic acid accounted for only 4.0% ± 0.1% of substrate content, but all but glucuronic acid were significantly different between substrates. These differences appeared to be largely driven by the smaller amounts of most polymers in cow dung (Fig. 3a) in combination with the small sample sizes (Table S10).

Statistical analyses of the polymer content of forage substrate, fungus comb, and guts were not done, because the samples were not comparable, but we were able to perform comparisons between fresh and old combs. These indicated that there was a total reduction in plant polymers from 38 to 46% of the total biomass in fresh combs to 30 to 44% in old combs (Fig. 3a). Again, cellulose, lignin, xylose, and glucose were the most abundant compounds, which were reduced from 13.7% ± 0.5%, 12.9% ± 0.5%, 6.7% ± 1.1%, and 4.0% ± 0.5% in fresh combs to 12.5% ± 1.6%, 11.6% ± 0.7%, 5.5% ± 0.6%, and 4.0% ± 0.3% in old combs (Fig. 3a). Old workers eat the old comb, and the polymer contents in guts were thus unsurprisingly further reduced to only 2.4% ± 1.1%, 4.0% ± 0.3%, 0.6% ± 0.1%, and 4.9% ± 0.3%, for cellulose, lignin, xylose, and glucose, respectively. Thus, while xylan and cellulose appeared to be most decomposed plant components within the comb, lignin was not greatly reduced [*F*_(1)_ = 9.559, *P* = 0.0029] before reaching old major worker guts. Glucose was the only monomer that increased in old major worker guts (Fig. 3a and Table S8).

The comprehensive microarray polymer profiling (CoMPP) analyses confirmed the overall pattern obtained by acid hydrolysis but provided a more detailed overview of changes in plant components (Fig. 3b and Table S11). A one-way analysis of variance (ANOVA) revealed a significant difference in polymer composition across termite species [*F*_(7)_ *=* 3.934, *P* = 0.0004]; however, the relative amounts of polysaccharides were not statistically significantly different between forage material and fresh combs [*F*_(10)_ *=* 0.76, *P* = 0.666]. On the other hand, the diversity of polysaccharides was higher in fungus combs than in forage material [*t*_(25)_ = −2.917, *P* = 0.007]. Hemicelluloses were abundant, and these polysaccharides were consumed during the decomposition process (Fig. 3b). While most plant components decreased from forage material to fungus comb and, subsequently, worker guts (Fig. 3b), some components (e.g., pectins) were present in greater amounts in fresh comb than in forage material, probably because other substrates were harvested more by these colonies than those included in our content analyses. As expected, pectins were either absent or present in very small amounts in termite guts [*F*_(7)_ *=* 3.934, *P* = 0.0004; Fig. 3b].

## DISCUSSION

### Plant biomass decomposition in the fungus-growing termite symbiosis.

The presence of a diverse assembly of plant-degrading enzymes, involving key glycoside hydrolases (GHs), AAs, CEs, and PLs, coded for by Termitomyces, underlines previous suggestions that the termite fungus can produce the enzymes necessary to decompose complex substrates (24). Their differential presence at different stages of the decomposition process highlights how the integrated combination of gut passage and an external fungal symbiont serves as an effective means to obtain very efficient plant biomass degradation.

It was first proposed in 1981 that the ingestion of nodules by termite workers was a strategy to increase the fungal load in the top part of the fungus comb (10). Later, this was confirmed to be important for substrate inoculation and to ensure monoculture farming through frequency-dependent selection of a single Termitomyces strain within a colony (39). It was also hypothesized that the first gut passage could facilitate enzyme transport to new substrates (“the ruminant hypothesis”) (29). Accordingly, Termitomyces spp. could use the first gut passage to efficiently move lignocellulosic enzymes from the old to the fresh comb. In our comparisons, fresh and old combs were distinct from each other and separated from guts and nodules (Fig. 2a), but normalization of enzyme activities indicated that this was not driven by differences in enzyme concentrations due to fungal content, because fresh comb became more similar to nodules and young worker guts than old comb in PCoA space (Fig. 2c). Although our enzyme assays cannot discriminate between fungal, termite, and bacterial enzymes expressed within guts, the most parsimonious explanation for the similarities in composition and activity of guts and nodules is that most of the enzymes that are active in guts are of fungal origin and originate from the exclusively fungal nodules (28, 29).

Our preliminary fungal RNA-seq indicated the expression of a wide range of Termitomyces enzymes in nodules, fresh comb, and old comb. This implies that even if our assertion that guts serve to transport enzymes is true, this transport is complemented by the expression of enzymes *in situ*. This combination of enzyme transport and *in situ* expression may be key to efficient boosting of plant biomass degradation after substrate inoculation, possibly a key innovation contributing to these insects becoming major decomposers in the Old World (7, 8).

### Which plant polymers are decomposed in the fungus-growing termite symbiosis?

Forage materials contained high concentrations of lignin, cellulose, and hemicellulose, most of which was depleted during the decomposition process. The high enzyme activities in guts could support previous suggestions that decomposition is initiated here (32, 40). However, the considerable amount of plant content in fresh combs suggests that guts are unlikely to be the prime location for decomposition. Recent work has suggested that gut passage in young Odontotermes formosanus workers could initiate lignin degradation (32). In contrast to that study, we found high lignin content and high expression of AAs targeting lignin in the fresh comb. Although we cannot rule out that this contrast is due to different methods applied, it could suggest that termite-Termitomyces species pairings differ in lignin processing. After old workers digest the old comb in a second gut passage, plant biomass is essentially completely degraded (28); this is consistent with our findings of very low polymer content in old worker guts, which did contain some plant polymers, as we sampled during the process of digestion and not from the excreted final feces, where all polymers are expected to have been utilized.

There was a highly significant decrease in the amount of cellulose content from forage material to the old worker termite guts, suggesting that Termitomyces spp. use cellulose as one of their main nutrient sources, and the transcriptome analysis showed the expression of a host of cellulose- and hemicellulose-degrading enzymes. The low expression of enzymes targeting pectin (polysaccharide lyases [PLs]) likely reflects that the substrate collected by the termites is mostly dead plant material with low pectin content. One of the most distinct changes in plant polymer amounts was in old termite guts, for which both plant polymer assays showed a substantial reduction in plant cell wall polymers, with only glucose increasing in concentration. Termitomyces spp. are able to grow on many carbon sources, but among the mono-, di-, and oligosaccharides, they grow best on cellobiose (24), and this was corroborated by the high expression of cellobiohydrolases in our transcriptomes. Gut microbes contain genes to break down fungus cell wall components and simple sugars, and since Termitomyces spp. do not grow well on glucose alone (24), the termites may obtain glucose when digesting old comb (see reference 41).

Our findings support the notion that the maintenance of an external fungal comb, inoculated with an optimal mix of plant material, fungal spores, and enzymes, is likely the key to the extraordinarily efficient plant decomposition in fungus-growing termites. The transcript analysis identified enzymes targeting not only all complex polysaccharides but also oxygen-dependent enzymes (e.g., LPMOs), supporting the idea that the comb serves as a versatile aerobic plant biomass conversion bioreactor. The efficiency of this bioreactor is likely comparable to decomposition in ruminants, but naturally, there are major differences between the two: the enzymes (functions and families) involved are markedly different, because biomass conversion is aerobic in the fungal comb. In the anaerobic rumen, biomass is converted to oligo-, di-, and monosaccharides that can be fermented to short-chain fatty acids and alcohols, which serve as nutrients for the host animal, while degraded plant components are primarily consumed by Termitomyces spp., with fungal biomass only later providing nutrition for the farming termites.

## MATERIALS AND METHODS

### Termite collections.

Samples were collected in 2015 and 2016 in South Africa at four geographical sites (Table 4). In 2015, old major workers, Termitomyces nodules, fresh comb, and old comb were obtained from three colonies from each of Odontotermes sp., Odontotermes cf. badius, and Macrotermes natalensis (Table 4). In 2016, both old and young workers, minor and major workers, Termitomyces nodules, and fresh and old combs were collected from one colony of Odontotermes sp. and three colonies of M. natalensis (Table 4). From each nest, approximately 100 mg of fungal comb and nodules was weighed, put into an 1.5-ml Eppendorf tube, and frozen at −80°C. Termites from the same nests were collected, and whole guts, including gut content, were dissected until 100 mg was obtained (typically 15 to 20 guts) and frozen at −80°C. To characterize plant polymer content in the forage harvested by fungus-growing termites, we collected and froze 50 to 200 g of forage at −20°C and sampled foraging termites for taxonomical identification from 18 foraging sites in 2016 (Table 1).

**TABLE 4:** Termite species, year of collection, geographical location, GenBank accession numbers for Odontotermes samples that needed identification, and biological replicates used the different experiments^*a*^

Termite species and colony code	Yr of collection	Location^*b*^	GenBank accession no.	GPS coordinates	Enzyme assays														Plant polymer content									RNA extraction			Fungal biomass measurement	
					AZCL							CPH							Lignin			Cellulose			Noncellulosic polymers							
					F.C.	O.C.	Nod.	O. mj. w.	O. mi. w.	Y. mj. w.	Y. mi. w.	F.C.	O.C.	Nod.	O. mj. w.	O. mi. w.	Y. mj. w.	Y. mi. w.	F.C.	O.C.	O. mj. w.	F.C.	O.C.	O. mj. w.	F.C.	O.C.	O. mj. w.	F.C.	O.C.	Nod.	F.C.	O.C.
Odontotermes sp. Od127	2015/2016	EF	KJ4590690 (Otani et al. [22])	−25.742700, 28.256517	X	X	X	X	X	X	X								X	X	X	X	X	X	X	X	X	X^*c*^	X^*c*^	X^*c*^	X	X
Odontotermes sp. Od128	2015	EF	KJ4590691 (Otani et al. [22])	−25.742400, 28.256617	X	X	X	X											X	X		X	X		X	X		X^*c*^	X^*c*^	X^*c*^	X	X
Odontotermes cf. badius Od145	2015	EF	MF092801	−25.751967, 28.258750	X	X	X	X											X	X		X	X		X	X		X	X	X	X	X
Odontotermes cf. badius Od150	2015	ARC	MF092802	−25.727767, 28.235200	X	X	X	X											X	X		X	X		X	X		X	X	X	X	X
Odontotermes cf. badius Od151	2015	ARC	MF092803	−25.727500, 28.235467	X	X	X	X											X	X		X	X			X		X	X	X	X	X
Odontotermes sp. Od159	2015	EF	MF092804	−25.747100, 28.255617	X	X	X	X											X	X		X	X		X	X		X	X	X	X	X
M. natalensis Mn156	2015	EF	NA	−25.743717, 28.260917	X	X	X	X											X	X		X	X		X	X		X	X	X	X	X
M. natalensis Mn160	2015	EF	NA	−25.742967, 28.260750	X	X	X	X											X	X		X	X		X	X		X	X	X	X	X
M. natalensis Mn162	2015	MO	NA	−24.661550, 28.792650	X	X	X	X	X	X	X	X	X	X	X	X	X	X	X	X		X	X		X	X		X	X	X	X	X
M. natalensis Mn154	2016	EF	NA	−25.743017, 28.260983	X	X	X	X											X	X	X	X	X	X	X	X	X				X	X
M. natalensis Mn164	2016	EF	NA	−25.746033, 28.257233	X	X	X	X	X	X	X	X	X	X	X	X	X	X	X	X	X	X	X	X	X	X	X				X	X
M. natalensis Mn173	2016	MO	NA	−24.661567, 28.793133	X	X	X	X	X	X	X	X	X	X	X	X	X	X	X	X	X	X	X	X	X	X	X				X	X

a X, samples from those nests were used in the given experiment; F.C., fresh comb; O.C., old comb; Nod, fungal nodules; O. mj. w., old major worker guts; O. mi. w., old minor worker guts; Y. mj. w., young major worker guts; Y. mi. w., young minor worker guts; NA, not available.

b EF, experimental farm; MO, Mookgophong; ARC, Rietondale.

c Samples that were RNA sequenced successfully.

### Termite identification.

Termites in the genus Odontotermes require molecular identification due to uncertainty in morphological identification (42). In contrast, M. natalensis is the only Macrotermes species reported in the sampling area (43), and molecular identification is thus not necessary. DNA extractions of three Odontotermes worker heads per colony were performed using the DNeasy blood and tissue kit (Qiagen, Hilden, Germany). The cytochrome oxidase II (COII) gene was amplified for comparison to available sequences in GenBank. PCR was prepared using the A-tLeu forward primer and B-tLys reverse primer (44). The PCR tube contained 8.5 μl of sterile distilled water, 1 μl of each primer, 2 μl of template, and 12.5 μl of REDTaq ReadyMix (Sigma-Aldrich, St. Louis, MO, USA). The conditions for PCR were as previously described (45). PCR products were visualized on agarose gels and purified using the MSB Spin PCRapace system (Stratec Molecular, Berlin, Germany). Purified PCR products were submitted to sequencing at Eurofins MWG Operon (Ebersberg, Germany). Sequences were aligned in Geneious Pro 4.8.5 using MUSCLE (46) and compared to Odontotermes COII sequences available from GenBank. A neighbor-joining tree was generated in MEGA 6.06 (47) with Kimura two-parameter estimates. The tree was built using termites sampled from the four Odontotermes nests involved in the enzyme and polymer content analyses (Table 4), eight samples from foraging sites (Table 1), and sequences described by Otani et al. (22) and from GenBank.

### Enzyme assays. (i) AZCL enzyme assays.

We conducted AZCL enzyme assays to determine enzyme activities at different stages of the decomposition process; old major worker guts, fungal nodules, fresh comb, and old comb from the 2015 collection, as well as young minor and major workers from 2016 were examined (Table 4). Seventeen AZCL polysaccharide media were prepared using 0.1 g/liter substrate in agarose medium (1% [wt/vol] agarose, 23 mM phosphoric acid, 23 mM acetic acid, 23 mM boric acid). For fungal comb measurements, the pH was adjusted separately for each substrate according to the manufacturer's description (Megazyme, Bray, Ireland) (De Fine Licht et al. [48]), and pH 6.0 was used for gut samples to mimic natural gut conditions (49). A 100-mg sample was crushed with a pestle in a 1.5-ml Eppendorf tube containing 1 ml of distilled water, vortexed, and centrifuged (15,000 × *g*). Fifteen microliters of supernatant was applied in triplicate in ca. 0.1-cm^2^ wells within the AZCL assay plates, which were photographed after 24 h of incubation at 25°C. Enzyme activity was inferred from measuring the halos around the wells using ImageJ version 1.6.0. (U.S. National Institutes of Health, Bethesda, MD, USA). Principal-coordinate analysis (PCoA) was performed in RStudio (50), and clustering analyses using Euclidean distance with bootstrap support after 10,000 permutations were performed in PAST version 2.17c (51).

### (ii) Enzyme screening using CPH substrates.

To validate the AZCL enzyme activity results, we used a new generation of versatile chromogenic substrates for high-throughput analysis of biomass-degrading enzymes provided by GlycoSpot (Frederiksberg C, Denmark) (Table S1) on samples from M. natalensis (Table 4). Briefly, 200 μl of activation solution was added per well, and plates were incubated for 15 min to activate the CPH substrates. The remaining activation solution was removed using a vacuum manifold at full pressure and washed twice with water to remove the stabilizer that keeps CPH substrates solid before they are washed. Samples were ground in 1 ml of 100 mM reaction buffer (50 mM sodium acetate [pH 5.0 for fungal samples and pH 6.0 for gut samples]). Five microliters of enzyme solution (Table S1) with a final enzyme concentration of 0.1 U/ml in the well for the positive control and 25-μl sample volumes were added as described by Kračun et al. (52). The plates were sealed using adhesive PCR seals (Thermo Scientific, VWR, Herlev, Denmark) and incubated for 23 h at 25°C at 150 rpm. After incubation, the liquid phase retained in the gel (reaction products) was separated by vacuum manifold, and the absorbance at 595 and 517 nm was measured in a plate reader for blue and red substrates, respectively (52).

### (iii) Enzyme activity relative to fungal content in fresh and old combs.

We tested whether the differences in enzyme activities in fresh versus old combs were due to differences in the amount of fungal material present, i.e., a question of concentration, or if fresh comb activities were more similar to their presumed origins (nodules via young worker guts). To do so, we estimated the relative amounts of fungal biomass in fresh and old combs. We did this by determining the amount of *N*-acetylglucosamine, which is deacetylated to glucosamine (GlcN) during hydrolysis, allowing for quantification by comparison to a standard curve of commercially available *N*-acetylglucosamine (Sigma-Aldrich, St. Louis, MO, USA) (53). We subsequently normalized the fresh-comb AZCL activities relative to fungal content and repeated the PCoA to evaluate the effect of this normalization on their relative positioning in PCoA space.

### RNA extraction, sequencing, and analyses. (i) RNA extraction and sequencing.

Approximately 20 mg of nodules, fresh comb, or old comb (Table 4) was placed in 1.5-ml Eppendorf tubes and frozen in liquid nitrogen within a few hours after sampling. Samples were ground with pestle to a fine powder. RNA was isolated using the RNeasy plant minikit (Qiagen, Hilden, Germany), according to the manufacturer's protocol. After RNA purity and quality were determined in a NanoDrop spectrophotometer (Thermo Scientific, Wilmington, DE, USA) and RNA yield and integrity analyses in Experion (Bio-Rad Laboratories, Hercules, CA, USA), only samples from M. natalensis Mn156 and Odontotermes sp. Od127 and Od128 were of sufficiently high quality for RNA-seq. mRNA was enriched by oligo(dT) beads to construct cDNA libraries, which were subsequently sequenced with 125-bp paired-end reads on the Illumina HiSeq 2500 platform.

### (ii) Transcript assembly and quantification.

Raw sequencing reads were first quality controlled, and reads were excluded if they contained more than 10% Ns or if their quality value was below 5 for more than 50% of the bases (Table S2). High-quality reads were *de novo* assembled using Trinity (version 2.3.2) (54), with default parameters. High-quality reads were then mapped to assembled transcripts using Bowtie2 (version 2.2.9) (55). To quantify the abundance of transcripts in each sample, reads mapped to transcripts were subsequently counted by RSEM (version 1.3.0) (56) to obtain transcripts per million (TPM) values of individual transcripts. The transcript sequences were deposited to the TSA database (Table S2).

### (iii) PPR and Hotpep.

Homology to Peptide Pattern (Hotpep [57]) with peptide pattern recognition (PPR) generated peptide patterns for all enzyme families in the Carbohydrate-Active enZymes (CAZy) database (58) was used to identify CAZymes in the translated transcripts, as previously described by Busk et al. (59). Briefly, Hotpep uses PPR-generated short conserved peptides (60) to assign protein sequences to specific groups within enzyme families. When functional information is available, as is the case for many of the carbohydrate-active enzymes, Hotpep uses this information to predict the functions of the annotated polypeptide sequences (58, 59).

### Plant polymer degradation. (i) Extraction and fractionation of plant cell wall material.

To determine plant polymer content, triplicate measurements were performed for forage substrate, fresh comb, old comb, and termite guts. Approximately 100 mg of material was freeze-dried and transferred to screw-cap plastic vials with stainless steel beads and ground to a fine powder. To extract the plant cell wall material (alcohol-insoluble residues [AIR]), a wash step in 70% (vol/vol) ethanol followed by a wash in methanol-chloroform (1:1 [vol/vol]) and an acetone extraction step were carried out to remove pigments, proteins, alkaloids, tannins, soluble sugars, and other low-molecular-weight metabolites. Samples were subsequently air-dried overnight before further processing (61, 62).

### (ii) CoMPP.

To look at structural polymer composition with high resolution, we performed CoMPP analyses using approximately 10 mg AIR (61). Samples were treated with an aqueous solution of cyclo-hexane-diamine-tetra-acetic acid (CDTA) to solubilize water-soluble cell wall components and Ca^2+^-chelated pectins, followed by an NaOH extraction to solubilize hemicellulose and cellulose. Four technical replicates, each in four different serial dilutions of the extracts/supernatants, were printed at room temperature and 55% humidity onto nitrocellulose membranes (Amersham Protran 0.45-μm nitrocellulose [NC] membranes) using a microarray printer (Sprint; Arrayjet, Roslin, UK). The arrays were incubated overnight in 5% (wt/vol) milk powder in phosphate-buffered saline (PBS) at 4°C for blocking to prevent antibodies from binding to the background (63). After blocking, the arrays were incubated in primary monoclonal antibodies (MAbs) for 2 h (64). After washing, the arrays were probed with secondary antibodies (Table S3) conjugated to alkaline phosphatase for 2 h before washing and developing in a BCIP-NBT (5-bromo-4-chloro-3-indolyphosphate–nitro-blue tetrazolium chloride) substrate (61). The developed microarrays were scanned at 2,400 dots per inch (dpi), and the signals were quantified using Array-Pro Analyzer 6.3 (Media Cybernetics, Rockville, MD, USA). An average signal intensity for three technical replicates and four dilutions was calculated, with the maximum value set to 100, and all other values were normalized accordingly. A heatmap was generated, and a cutoff of 5 was imposed to avoid false positives due to background signal (62, 65).

### Lignin.

Twenty milligrams of AIR per sample was hydrolyzed with 25% (vol/vol in glacial acetic acid) acetyl bromide at 70°C for 30 min (66). After incubation for complete digestion, the samples were cooled in an ice bath, mixed with 0.9 ml of 2 M NaOH and 0.1 ml of 5 M hydroxylamine-HCl, and finally, 6 ml of glacial acetic acid was added to complete lignin solubilization. The samples were centrifuged at 1,400 × *g* for 5 min, supernatants were collected, and the absorbance at 280 nm was measured (67). A standard curve was built with alkali lignin (Sigma-Aldrich, St. Louis, USA), and the absorptivity value (ε) was 22.9 g^−1^ · liter · cm^−1^.

### Cellulose and noncellulosic monomers.

Five milligrams of AIR was hydrolyzed for 1 h at 120°C using 300 μl of 2 M trifluoroacetic acid (TFA), after which samples were spun down at maximum speed and ethanol was added until a concentration of 70% (vol/vol) was obtained. The tubes were centrifuged at 10,000 × *g*, the ethanol was removed, and the samples were air-dried for 1 h at room temperature. Three hundred microliters of 72% (vol/vol) sulfuric acid was added, and samples were incubated for 1 h at room temperature, after which 55 μl of sample was added to 945 μl of water to reach a 4% (vol/vol) sulfuric acid concentration. Samples were incubated at 120°C for 1 h; after this, the tubes were centrifuged at 10,000 × *g*, the supernatant was retrieved and diluted 50 times before quantification with high-performance anion-exchange chromatography with pulsed amperometric detection (HPAEC-PAD) using a Dionex ICS 5000^+^ DC system equipped with a 4-μm SA-10 column with 2 by 250-mm dimensions and a precolumn. The run conditions were 0.3 ml/min, column temperature of 40°C, and 1 mM NaOH eluent for 0 to 8 min followed by 100 mM NaOH for up to 20 min with 10 min of subsequent equilibration in 1.0 mM NaOH. To measure noncellulosic monomers, a 3- to 5-mg AIR sample was hydrolyzed with 800 μl of 2 M TFA for 1 h at 120°C (68). After incubation, samples were cooled in an ice bath and centrifuged at 10,000 × *g*, and the TFA was removed by evaporation under vacuum overnight. The hydrolysis products were resuspended in 300 μl of deionized water (68). Samples were further diluted 20 times in Milli-Q water before quantification of monosaccharide constituents by HPAEC-PAD as described above.

### Statistical analyses.

Table S4 provides an overview of the statistical analyses performed and their results. The assays were evaluated separately, analyses were performed in R version 3.3.2 (50), and *P* values were Bonferroni corrected for multiple comparisons. The hist function in R was used to test if response variables had equal variance; if so, we used linear mixed models (LMM), with colony component (nodules, guts, old comb, and fresh comb), species (M. natalensis, Odontotermes sp., and Odontotermes cf. badius), and year (2015 and 2016) as fixed variables and colony origin as a random variable. We tested if different plant substrates differed in lignin and cellulose content, but since colony information was not available for these tests, we used linear models (LM) with foraging substrate and termite species as fixed factors. For each model, one-way nested ANOVAs were used to determine the effects of the fixed factors. Pilot experiments (data not shown) indicated a linear association between concentration of enzyme extracts and halo areas. Thus, for AZCL and CPH substrate variables with unequal variance (Table S4), we conducted nonparametric Kruskal-Wallis rank sum tests. For the remaining AZCL and CPH substrates, we conducted one-way nested ANOVAs fitted on linear mixed models. To evaluate the diversity of the AZCL enzymes, we determined Shannon indices, which were transformed exponentially to ensure equal variances, and subsequently fitted a linear model using the Shannon values as the response variable and colony component (guts, fresh comb, nodules, and old comb) and termite species as fixed factors.

For noncellulosic monomer, cellulose, and lignin content measurements with equal variances, we used LMM to test for differences between colony components, while we used Kruskal-Wallis rank sum tests for variables with unequal variance. Similarly, we employed LM to test for differences between forage types for contents with equal variance and Kruskal-Wallis rank sum tests for variables with unequal variance (Table S4). For the CoMPP data, we employed one-way ANOVAs, followed by unpaired Student's *t* tests, with absorbance as the response variable and substrate, old worker guts, fresh comb, and old comb as main factors.

### Accession number(s).

Sequence data were deposited to the SRA database with accession numbers SRR5944781 to SRR5944786 and SRR5944350 to SRR5944352 (Table S2).

## Supplementary Material

Supplemental material
