# Supplementary material for: Enzyme Activities at Different Stages of Plant Biomass Decomposition in Three Species of Fungus-Growing Termites
Source: Appl Environ Microbiol. 2018 Feb 14;84(5):e01815-17. doi: 10.1128/AEM.01815-17 (PMC5812949; doi:10.1128/AEM.01815-17)
Supplement: Supplemental material [file supp_84_5_e01815-17__index.html]

Supplemental material 

# Enzyme Activities at Different Stages of Plant Biomass Decomposition in Three Species of Fungus-Growing Termites

## Supplemental material

- Supplemental file 1 -

  Chromogenic polysaccharide hydrogel substrates, their sources, and positive controls for enzymatic reactions (Table S1); reads and base numbers obtained from the transcriptomes from one *Macrotermes natalensis* colony and two *Odontotermes* sp. colonies (Table S2); list of antibodies used to probe microarrays, their binding specificities, and publication origins (Table S3); statistical analyses and their results (Table S4); predicted enzyme families (CAZymes) and expression level of transcript sequences represented in the Termitomyces transcriptomes in different sites of the decomposition process in *Macrotermes natalensis* and *Odontotermes* sp. (Table S5); expression level and distribution of transcripts across different sites of the decomposition process in one *Macrotermes natalensis* colony and two *Odontotermes* sp. colonies (Table S6); AZCL enzyme activities in different samples collected in 2015 and 2016 after 24 h of incubation (Table S7); enzyme activities in *Macrotermes natalensis* colony components detected after 24 h of incubation of extracts with chromogenic polysaccharide hydrogel (Table S8); normalization of AZCL enzyme activities in fresh comb samples (Table S9); polysaccharide content of AIR sample (Table S10); comprehensive microarray polymer profiling heat map (Table S11); phylogenetic analysis placing the *Odontotermes* COII sequences from foraging sites and nests used for the enzyme, polymer content, and RNAseq in a phylogeny with reference *Odontotermes* sequences (Fig. S1); comparison of CPH and AZCL substrate enzyme screening (Fig. S2).

  PDF, 1.7M
