# Supplementary material for: Enzyme Activities at Different Stages of Plant Biomass Decomposition in Three Species of Fungus-Growing Termites
Source: Appl Environ Microbiol. 2018 Feb 14;84(5):e01815-17. doi: 10.1128/AEM.01815-17 (PMC5812949; doi:10.1128/AEM.01815-17)
Supplement: Supplemental material [file AEM.01815-17_zam005188351s1.pdf]

Supplemental Material

for

# Enzyme activities at different stages of plant biomass decomposition in three species of fungus-farming termites

Rafael R. da Costa[1], Haofu Hu[1], Bo Pilgaard[2], Sabine M.E. Vreeburg [3], Julia Schückel[4], Kristine S.K. Pedersen[1], Stjepan K. Kračun[4], Peter K. Busk[2], Jesper Harholt[5], Panagiotis Sapountzis[1], Lene Lange[2], Duur K. Aanen[3] and Michael Poulsen[1]

Table S1

Table S2

Table S3

Table S4

Table S5

Table S6

Table S7

Table S8

Table S9

Table S10

Table S11

Figure S1

Figure S2

Supplemental References

| Substrate | Source | Enzymes used as positive control | Enzyme code |
|---|---|---|---|
| *Starch* | | | |
| CPH-amylose* | Potato | α-amylase | eMA3 |
| *Pectin* | | | |
| CPH-rhamnogalacturonan | Soy bean | rhamnogalacturonanase | ePEC1 |
| CPH-galactomannan | Carob | endo-β-1,4-mannanase (*Cellvibriojaponicus*) | eMAN2 |
| *Cellulose* | | | |
| CPH-2-hydroxyethylcellulose (CPH-HE cellulose) | N/A | endo-cellulase (*Trichoderma longibrachiatum*) | eCEL1 |
| CPH-glucan from barley | Barley | endo-β-1,3-glucanase | eGLC1 |
| *1,3-Glucans* | | | |
| CPH-pachyman | *Poriacocos* | endo-β-1,3-glucanase | eGLC1 |
| CPH-curdlan | *Alcaligene faecalis* | endo-β-1,3-glucanase | eGLC1 |
| *Cross-linking Glycans* | | | |
| CPH-xylan | Beechwood | endo-β-1,4-xylanase (*Aspergillus niger*) | eXYL1 |
| CPH-arabinoxylan | Wheat | endo-β-1,4-xylanase (*Aspergillus niger*) | eXYL1 |
| CPH-xyloglucan | Tamarind | Xyloglucanase (*Paenibacillus* sp.) | eXG1 |

Table S1: Chromogenic polysaccharide hydrogel (CPH) substrates, their sources and positive controls for enzymatic reactions (Kračun et al 2015). All enzymes used as positive control were provided by Megazyme, except for α-amylase which was purchased from Sigma Aldrich.

| Nest | SRA accession # | TSA accession # | Sample origin | # of raw reads | # of clean reads | Raw reads (Gbp) | Clean reads (Gbp) | Q20 (%) | Total transcript length (Mbp) | Transcript number |
|---|---|---|---|---|---|---|---|---|---|---|
| *Macrotermes natalensis* Mn156 | SRR5944782 | GFVP00000000 | Nodules | 18,495,712 | 18,038,605 | 4.62 | 4.51 | 95.2 | 113.2 | 46,907 |
| | SRR5944783 | GFVQ00000000 | Old comb | 16,531,848 | 16,220,236 | 4.13 | 4.06 | 95.4 | 66.61 | 39,664 |
| | SRR5944781 | GFVO00000000 | Fresh comb | 14,573,164 | 14,181,014 | 3.64 | 3.55 | 95.1 | 100.1 | 53,438 |
| *Odontotermes* sp. (Od127) | SRR5944352 | GFVS00000000 | Nodules | 17,095,396 | 16,685,151 | 4.27 | 4.17 | 95.3 | 102.0 | 171,011 |
| | SRR5944351 | GFVR00000000 | Fresh comb | 14,515,681 | 14,252,820 | 3.63 | 3.56 | 95.0 | 87.17 | 42,168 |
| | SRR5944350 | GFVT00000000 | Old comb | 15,606,642 | 15,096,393 | 3.9 | 3.77 | 94.7 | 72.52 | 45,835 |
| *Odontotermes* sp. (Od128) | SRR5944785 | GFVU00000000 | Nodules | 17,657,192 | 17,231,003 | 4.41 | 4.31 | 95.2 | 119.1 | 107,656 |
| | SRR5944784 | GFVV00000000 | Fresh comb | 14,526,284 | 14,239,562 | 3.63 | 3.56 | 95.3 | 60.79 | 57,960 |
| | SRR5944786 | GFVW00000000 | Old comb | 16,679,849 | 16,228,702 | 4.17 | 4.06 | 95.0 | 72.47 | 40,617 |

Table S2: Reads and base numbers obtained from the transcriptomes from one *Macrotermes natalensis* colony and two *Odontotermes* sp. colonies.

| **Monoclonal antibody code** | **Specificity** | **Reference** |
|---|---|---|
| BS-400-2 | β-(1→3) D-glucan | Meikle et al., (1991) |
| BS-400-3 | β- (1→3)(1→4) D-glucan | Meikle et al., (1994) |
| BS-400-4 | β- (1→4) D-mannan | Pettolino et al., (2001) |
| CBM3a | Cellulose | Tormo et al., (1996) |
| LM1 | Extensin | Smallwood et al., (1995) |
| LM2 | Arabinogalactan protein | Yates et al., (1996) |
| LM5 | β- (1→4) D-galactan | Jones et al., (1997) |
| LM6 | α-(1→5) L-arabinan | Willats et al., (1998) |
| LM7 | Homogalactauronan with an intermediate DE, non-blockwise distribution of MeOH | Willats et al., (2001) |
| LM8 | Xylogalacturan | Willats et al., (2004) |
| LM10 | (1→4)-β-D-xylan | McCartney et al., (2005) |
| LM11 | Xylan/arabinoxylan | McCartney et al., (2005) |
| LM13 | Arabinan | Moller et al., (2008) |
| LM15 | Xyloglucan | Marcus et al., (2008) |
| LM16 | Arabinan | Verhertbruggen et al., (2009) |
| LM18 (MUC2) | Homogalacturonan | Verhertbruggen et al., (2009) |
| LM19 (XGA2) | Homogalacturonan | Verhertbruggen et al., (2009) |
| LM20 (MUC1) | Homogalacturonan | Verhertbruggen et al., (2009) |
| LM21 | Mannan | Marcus et al., (2010) |
| LM22 | Mannan | Marcus et al., (2010) |
| LM23 | β-(1→4) D-xylan | McCartney et al., (2005) |
| LM24 | Xyloglucan | Pedersen et al., (2012) |
| LM25 | Xyloglucan | Pedersen et al., (2012) |
| JIM5 | Homogalacturonan with low degree of esterification, partially methyl esterified (Low DE) | Clausen et al., (2003) |
| JIM6 | No defined epitope (Anti callose/MLG like binding) | Clausen et al., (2003) |
| JIM7 | Homogalacturonan, partially methyl esterified | Clausen et al., (2003) |

Table S3: List of antibodies used to probe microarrays, their binding specificities, and publication origins

| Experiment | Statistical analyses | Variable | Factors | Test statistic | df | P |
|---|---|---|---|---|---|---|
| **AZCL** | One-way nested ANOVA fitted on linear mixed models (equal variance) | Arabinoxylan | Colony component | F = 16.89 | 6 | <0.0001 |
| | | Casein | | F = 95.73 | 6 | <0.0001 |
| | | He-cellulose | | F = 26.52 | 6 | <0.0001 |
| | | Xylan | | F = 11.38 | 6 | <0.0001 |
| **AZCL** | One-way nested ANOVA fitted on linear mixed models (equal variance) | Arabinoxylan | Termite species | F = 2.494 | 2 | 1.000 |
| | | Casein | | F = 9.278 | 2 | 0.7670 |
| | | He-cellulose | | F = 10.95 | 2 | <0.0001 |
| | | Xylan | | F = 3.124 | 2 | 1.000 |
| **AZCL** | Kruskal-Wallis rank sum test (unequal variance) | Amylose | Colony component | $\chi^2$ = 165.0 | 6 | <0.0001 |
| | | Barley β-glucan | | $\chi^2$ = 76.80 | 6 | <0.0001 |
| | | Collagen | | $\chi^2$ = 84.44 | 6 | <0.0001 |
| | | Curdlan | | $\chi^2$ = 98.51 | 6 | <0.0001 |
| | | Debranched Arabinan | | $\chi^2$ = 15.05 | 6 | 0.2579 |
| | | Galactan | | $\chi^2$ = 58.46 | 6 | <0.0001 |
| | | Galactomannan | | $\chi^2$ = 63.77 | 6 | <0.0001 |
| | | Rhamnogalacturonan | | $\chi^2$ = 39.02 | 6 | <0.0001 |
| | | Xyloglucan | | $\chi^2$ = 39.63 | 6 | 0.0065 |
| **AZCL** | Kruskal-Wallis rank sum test (unequal variance) | Amylose | Termite species | $\chi^2$ = 3.899 | 2 | 1.000 |
| | | Barley-β-glucan | | $\chi^2$ = 2.710 | 2 | 1.000 |
| | | Collagen | | $\chi^2$ = 33.08 | 2 | <0.0001 |
| | | Curdlan | | $\chi^2$ = 47.50 | 2 | <0.0001 |
| | | Debranched Arabinan | | $\chi^2$ = 8.956 | 2 | 0.1476 |
| | | Galactan | | $\chi^2$ = 41.60 | 2 | <0.0001 |
| | | Galactomannan | | $\chi^2$ = 60.51 | 2 | <0.0001 |
| | | Rhamnogalacturonan | | $\chi^2$ = 13.28 | 2 | 0.0169 |
| | | Xyloglucan | | $\chi^2$ = 75.32 | 2 | <0.0001 |
| **AZCL** | One-way nested ANOVA fitted on linear mixed models | Exponentially transformed Shannon indices | Colony component | F = 52.86 | 6 | <0.0001 |
| | | | Termite species | F = 8.427 | 2 | 0.1144 |
| **CPH** | One-way nested ANOVA fitted on linear mixed models (equal variance) | Amylose | Colony component | F = 4.318 | 6 | 0.0156 |
| | | Arabinoxylan | | F = 15.13 | 6 | <0.0001 |
| | | Barley-β-glucan | | F = 25.42 | 6 | <0.0001 |
| | | Curdlan | | F = 23.80 | 6 | <0.0001 |
| | | Galactomannan | | F = 3.227 | 6 | 0.0870 |
| | | He-cellulose | | F = 12.27 | 6 | <0.0001 |
| **CPH** | Kruskal-Wallis rank sum test (unequal variance) | Pachyman | Colony component | $\chi^2$ = 40.97 | 6 | 0.0029 |
| | | Rhamnogalacturonan | | $\chi^2$ = 17.24 | 6 | 0.0840 |
| | | Xylan | | $\chi^2$ = 45.39 | 6 | <0.0001 |

| | | | | | | |
|---|---|---|---|---|---|---|
| | | Xyloglucan | | $\chi^2$ = 9.353 | 6 | 1.000 |
| **Non-cellulosic polymers** | One-way nested ANOVA fitted on linear mixed models (equal variance) | Arabinose | Colony component | F = 20.86 | 1 | <0.0001 |
| | | Galactose | | F = 2.039 | 1 | 0.1569 |
| | | Glucose | | F = 0.143 | 1 | 0.7059 |
| | | Xylose | | F = 9.955 | 1 | 0.0022 |
| | | Mannose | | F = 0.010 | 1 | 0.9189 |
| | | Galacturonic acid | | F = 2.818 | 1 | 0.0968 |
| | | Fucose | | F = 0.010 | 1 | 0.9194 |
| | | Glucuronic acid | | F = 17.33 | 1 | <0.0001 |
| **Non-cellulosic polymers** | One-way nested ANOVA fitted on linear mixed models (equal variance) | Arabinose | Termite species | F = 4.907 | 2 | 0.0174 |
| | | Galactose | | F = 4.602 | 2 | 0.0185 |
| | | Glucose | | F = 7.279 | 2 | 0.0002 |
| | | Xylose | | F = 10.53 | 2 | 0.0004 |
| | | Mannose | | F = 3.674 | 2 | 0.0546 |
| | | Galacturonic acid | | F = 4.222 | 2 | 0.0224 |
| | | Fucose | | F = 0.267 | 2 | 0.8476 |
| | | Glucuronic acid | | F = 1.429 | 2 | 0.2770 |
| **Non-cellulosic polymers** | One-way nested ANOVA fitted on linear mixed models (equal variance) | Arabinose | Year | F = 3.562 | 1 | 0.0629 |
| | | Galactose | | F = 28.18 | 1 | <0.0001 |
| | | Glucose | | F = 32.39 | 1 | <0.0001 |
| | | Xylose | | F = 0.051 | 1 | 0.8214 |
| | | Mannose | | F = 10.62 | 1 | 0.0020 |
| | | Galacturonic acid | | F = 1.478 | 1 | 0.2279 |
| | | Fucose | | F = 0.069 | 1 | 0.7931 |
| | | Glucuronic acid | | F = 0.0005 | 1 | 0.9821 |
| **Cellulose** | One-way nested ANOVA fitted on linear mixed models (equal variance) | Cellulose content | Colony component | F = 0.9655 | 1 | 0.3320 |
| | | | Termite species | F = 4.459 | 2 | 0.0412 |
| **Lignin** | One-way nested ANOVA fitted on linear mixed models (equal variance) | Lignin content | Colony component | F = 9.559 | 1 | 0.0029 |
| | | | Termite species | F = 0.2131 | 2 | 0.8116 |
| **Cellulose in the forage material** | Type III ANOVA tests of a linear model (equal variances) | Cellulose | Forage substrate types | F = 8.822 | 3 | 0.0002 |
| | | | Termite species | F = 6.032 | 2 | 0.0062 |
| **Lignin in the forage material** | Type III ANOVA test of a linear model (equal variances) | Lignin content | Forage substrate types | F = 0.170 | 3 | 0.5424 |
| | | | Termite species | F = 0.307 | 2 | 0.1563 |
| **Non-cellulosic polymers in the forage material** | Type III ANOVA test of a linear model (equal variances) | Glucose | Forage substrate types | F = 12.41 | 3 | <0.0001 |
| | | Xylose | | F = 6.954 | 3 | 0.0005 |
| | | Mannose | | F = 18.44 | 3 | <0.0001 |
| | | Glucuronic acid | | F = 1.270 | 3 | 0.2957 |

| | | | | | | |
|---|---|---|---|---|---|---|
| | | Galacturonic acid | | F = 12.43 | 3 | <0.0001 |
| **Non-cellulosic polymers in the forage material** | ANOVA type III test of a linear model (equal variances) | Glucose | Termite species | F = 0.5176 | 2 | 0.5993 |
| | | Xylose | | F = 1.683 | 2 | 0.1964 |
| | | Mannose | | F = 3.480 | 2 | 0.0380 |
| | | Glucuronic acid | | F = 2.326 | 2 | 0.0103 |
| | | Galacturonic acid | | F = 3.104 | 2 | 0.0545 |
| **Non-cellulosic polymers in the forage material** | Kruskal-Wallis rank sum test (unequal variances) | Fucose | Forage substrate types | $\chi^2 = 21.24$ | 3 | <0.0001 |
| | | Arabinose | | $\chi^2 = 15.73$ | 3 | 0.0013 |
| | | Galactose | | $\chi^2 = 18.47$ | 3 | 0.0003 |
| **Non-cellulosic polymers in the forage material** | Kruskal-Wallis rank sum test (unequal variances) | Fucose | Termite species | $\chi^2 = 19.98$ | 2 | <0.0001 |
| | | Arabinose | | $\chi^2 = 55.13$ | 2 | 0.0635 |
| | | Galactose | | $\chi^2 = 1.304$ | 2 | 0.5210 |

Table S4: All statistical analyses and their results. Colony components for AZCL and CPH = old major and minor worker guts, young major and minor worker guts, fresh comb, old comb, nodules; Colony components for Non-cellulosic polymers, Cellulose and Lignin = fresh and old comb; termite species = *M. natalensis*, *O.* cf. *badius*, and *O.* sp.

| Class of enzymes | EC # | Functional description | Expression level (Transcript per Million) | | | | | | | | | # of transcript sequences placed in different CAZy families | | | | | | | | |
|---|---|---|---|---|---|---|---|---|---|---|---|---|---|---|---|---|---|---|---|---|
| | | | *Macrotermes natalensis* 156 | | | *Odontotermes* sp. | | | | | | *Macrotermes natalensis* 156 | | | *Odontotermes* sp. | | | | | |
| | | | Nodules | Fresh comb | Old comb | Nodules (Od127) | Fresh comb (Od127) | Old comb (127) | Nodules (Od128) | Fresh comb (Od128) | Old comb (Od128) | Nodules | Fresh comb | Old comb | Nodules (Od127) | Fresh comb (Od127) | Old comb (127) | Nodules (Od128) | Fresh comb (Od128) | Old comb (Od128) |
| Auxiliary Activities | 1.1.3.10 | pyranose oxidase | 1.440 | 7.940 | 12.57 | - | - | 22.81 | 5.200 | - | 15.41 | 1 AA3 | 3 AA3 | 2 AA3 | - | - | 3 AA3 | 4 AA3 | - | 11 AA3 |
| | 1.1.3.13 | alcohol oxidase | 307.9 | 191.1 | 2006 | 0.490 | 1920 | 161.1 | 67.13 | 1103 | 19.00 | 17 AA3 | 15 AA3 | 6 AA3 | 1 AA3 | 24 AA3 | 3 AA3 | 21 AA3 | 5 AA3 | 19 AA3 |
| | 1.1.99.18 | cellobiose dehydrogenase (acceptor) | 196.0 | 184.9 | 302.1 | 46.79 | 635.7 | 259.9 | 166.1 | 84.80 | 96.73 | 12 AA3,3 AA8 | 7 AA3,3 AA8 | 5 AA3,3 AA8 | 8 AA3 | 7 AA3,1 AA8 | 12 AA3,1 AA8 | 19 AA3,2 AA8 | 7 AA3,1 AA8 | 16 AA3,2 AA8 |
| | 1.1.99.29 | pyranose dehydrogenase (acceptor) | 13.77 | 36.08 | 8.600 | - | 30.27 | 7.200 | 13.88 | 27.29 | 4.790 | 4 AA3 | 18 AA3 | 4 AA3 | - | 8 AA3 | 3 AA3 | 2 AA3 | 8 AA3 | 1 AA3 |
| | 1.10.3.2 | laccase | 516.4 | 1463 | 1902 | 63.29 | 2536 | 748.0 | 1637 | 4605 | 478.30 | 134 AA1 | 86 AA1 | 45 AA1 | 19 AA1 | 69 AA1 | 61 AA1 | 78 AA1 | 43 AA1 | 69 AA1 |
| | NA | LPMO -expanded families | 600.8 | 197.5 | 177.3 | 30.10 | 677.3 | 580.3 | 163.7 | 351.4 | 2257 | 21 AA9 | 18 AA9 | 16 AA9 | 23 AA9,1 AA10 | 13 AA9 | 23 AA9 | 29 AA9,1 AA11 | 35 AA9,6 AA11 | 41 AA9 |
| | 1.11.1.11 | L-ascorbate peroxidase | - | - | - | - | - | - | - | - | - | - | - | - | - | - | - | - | - | - |
| | 1.11.1.16 | versatile peroxidase | 2070 | 542.2 | 95.25 | - | 1317 | 66.78 | 1.050 | 4.320 | 0.950 | 2 AA2 | 1 AA2 | 1 AA2 | - | 1 AA2 | 2 AA2 | 1 AA2 | 1 AA2 | 1 AA2 |
| | | Sum | **1 638** | **2 623** | **4 504** | **140.7** | **7 116** | **1 846** | **2 054** | **6 176** | **2 872** | **194** | **151** | **82** | **52** | **123** | **108** | **157** | **106** | **140** |
| Polysaccharide Lyases | 4.2.2.* | | 57.96 | 58.57 | 84.37 | 5.910 | 108.0 | 90.03 | 42.93 | 10.02 | 49.58 | 18 PL4 | 12 PL4 | 4 PL4 | 4 PL4 | 31 PL4 | 2 PL4 | 8 PL4 | 10 PL4 | 6 PL4 |
| | 4.2.2.2 | pectate lyase | 100.7 | 239.8 | 191.9 | 8.620 | 312.2 | 77.29 | 237.3 | 26.36 | 33.64 | 8 PL1,19 PL3 | 28 PL3 | 13 PL1,9 PL3 | 1 PL1,1 PL3 | 12 PL1,9 PL3 | 12 PL1,5 PL3 | 4 PL1, | 1 PL1, | 5 PL1, |
| | 4.2.2.3 | poly(β-D-mannuronate) lyase | 579.5 | 1024 | 932.2 | 7.330 | 1271 | 94.49 | 113.8 | 94.24 | 100.2 | 4 PL14 | 2 PL14 | 2 PL14 | 1 PL14 | 2 PL14 | 5 PL14 | 6 PL14 | 5 PL14 | 5 PL14 |
| | | Sum | **738.1** | **1322** | **1208** | **21.86** | **1 691.09** | **261.81** | **394.02** | **130.62** | **183.41** | **49** | **42** | **28** | **7** | **54** | **24** | **23** | **21** | **22** |
| Carbohydrate esterases | 3.1.1.* | | 40.99 | 67.51 | 175.4 | 3.530 | 157.4 | 275.4 | 109.8 | 139.3 | 340.5 | 17 CE12 | 10 CE12 | 5 CE12 | 1 CE12 | 30 CE12 | 5 CE12 | 12 CE12,1 CE13 | 8 CE12,1 CE15 | 3 CE12 |
| | 3.1.1.1 | carboxylesterase | 65.90 | 73.99 | 69.57 | 50.91 | 96.32 | 259.9 | 63.34 | 193.3 | 153.0 | 3 CE0 | 1 CE0 | 2 CE0 | 3 CE0 | 6 CE0 | 8 CE0 | 28 CE0 | 14 CE0 | 3 CE0 |
| | 3.1.1.6 | acetylesterase | 30.75 | 121.5 | 251.1 | 0.00 | 122.3 | 40.95 | 253.7 | 41.65 | 45.73 | 5 CE16 | 4 CE16 | 3 CE16 | - | 3 CE16 | 3 CE16 | 4 CE16 | 5 CE16 | 3 CE16 |
| | 3.1.1.11 | pectinesterase | 28.17 | 96.87 | 98.62 | 0.95 | 232.9 | 65.65 | 21.00 | 18.22 | 74.66 | 23 CE8 | 26 CE8 | 10 CE8 | 1 CE8 | 15 CE8 | 15 CE8 | 13 CE8 | 5 CE8 | 7 CE8 |
| | 3.1.1.72 | Acetylxylan esterase | 142.4 | 235.1 | 488.1 | 4.45 | 799.17 | 230.29 | 217.09 | 145.34 | 775.65 | 6 CE1,10 CE4 | 4 CE1,7 CE4 | 2 CE1,4 CE4 | 2 CE1,1 CE6 | 4 CE1,11 CE4 | 3 CE1,6 CE4 | 2 CE1,13 CE4 | 4 CE1,5 CE4 | 5 CE1,16 CE4 |
| | 3.1.1.73 | feruloyl esterase | - | - | - | 1.10 | - | - | - | - | - | - | - | - | 3 CE1 | - | - | - | - | - |
| | 3.1.1.74 | cutinase | 9.040 | 63.86 | 120.1 | - | 186.0 | - | - | - | - | 3 CE5 | 8 CE5 | 2 CE5 | - | 5 CE5 | - | - | - | - |
| | 3.5.1.* | | NA | NA | NA | 0.70 | NA | NA | NA | NA | NA | 8 CE4 | 6 CE4 | 4 CE4 | 1 CE11 | 3 CE4 | 11 CE4 | 6 CE4 | 3 CE4 | 22 CE4 |
| | 3.5.1.25 | N-acetylglucosamine-6-phosphate deacetylase | - | - | - | 2.49 | - | - | - | - | - | - | - | - | 2 CE9 | - | - | - | - | - |
| | 3.5.1.41 | chitin deacetylase | 315.2 | 989.2 | 490.3 | 15.60 | 1378.4 | 611.0 | 735.7 | 537.8 | 703.5 | 8 CE4 | 6 CE4 | 4 CE4 | 5 CE4 | 3 CE4 | 11 CE4 | 6 CE4 | 3 CE4 | 22 CE4 |

| | | | | | | | | | | | | | | | | | | | | |
|---|---|---|---|---|---|---|---|---|---|---|---|---|---|---|---|---|---|---|---|---|
| | 3.5.1.89 | N-acetylglucosaminylphosphatidylinositol deacetylase | - | - | - | 3.060 | - | - | - | - | - | - | - | - | 1 CE14 | - | - | - | - | - |
| | | Sum | **1638.36** | **9538.55** | **13117.32** | **407.85** | **20587.80** | **5698.97** | **6295.93** | **13689.05** | **8204.07** | **83** | **72** | **36** | **20** | **80** | **62** | **85** | **48** | **81** |
| **Glycoside Hydrolases** | 2.4.1.* | | NA | NA | NA | 4.250 | 30.19 | 18.88 | 1.670 | 44.53 | 5.950 | 2 GH13 | 2 GH13 | 1 GH13 | 1 GH72 | 1 GH16 | 2 GH16,3 GH72 | 1 GH16 | 3 GH16,16 GH72 | 1 GH16 |
| | 2.4.1.18 | 1,4-α-glucan branching enzyme | 32.53 | 40.33 | 47.13 | 26.64 | 35.70 | 57.79 | 27.99 | 48.04 | 49.21 | 2 GH13 | 2 GH13 | 1 GH13 | 7 GH13 | 1 GH13 | 2 GH13 | 3 GH13 | 2 GH13 | 1 GH13 |
| | 2.4.1.25 | 4-α-glucanotransferase | 97.98 | 99.06 | 111.5 | 23.77 | 88.68 | 47.82 | 20.51 | 39.18 | 67.92 | 6 GH13,6 GH133 | 2 GH13,23 | 3 GH13,3 GH133 | 6 GH13,6 GH133,2 GH77 | 1 GH13,1 GH133 | 2 GH13,2 GH133 | 3 GH13,3 GH133 | 2 GH13,2 GH133 | 2 GH13,2 GH133 |
| | 2.4.1.183 | α-1,3-glucan synthase | 87.67 | 201.3 | 143.3 | - | 152.1 | 42.50 | 13.70 | 30.38 | 30.58 | 6 GH13 | 2 GH13 | 6 GH13 | - | 5 GH13 | 1 GH13 | 1 GH13 | 3 GH13 | 3 GH13 |
| | 2.4.1.207 | xyloglucan:xyloglucosyl transferase | - | - | 2.340 | - | - | - | 5.670 | 0.000 | 3.630 | - | - | 1 GH16 | - | - | - | 3 GH16 | - | 1 GH16 |
| | 3.2.1.* | | 124.0 | 142.3 | 64.27 | 8.780 | 55.87 | 91.89 | 27.06 | 10.90 | 20.85 | 2 GH125,9 GH131,6 GH35,26 GH92 | 5 GH125, 11GH13 1,1 GH132,4 GH35,19 GH92 | 6 GH131,7 GH35,17 GH92 | GH73,4 GH92 | 6 GH125,1 3 GH131,2 GH92 | 1 GH125,3 GH131,1 GH132,3 GH35,16 GH92 | 2 GH131,1 GH35,10 GH92 | 5 GH131,1 GH88,2 GH92 | 1 GH131,1 2 GH92 |
| | 3.2.1.1 | α-amylase | 98.25 | 156.29 | 167.70 | 16.23 | 132.0 | - | 26.01 | 14.71 | 13.94 | 15 GH13 | 12 GH13 | 3 GH13 | 3 GH13,1 GH57 | 15 GH13 | - | 4 GH13 | 5 GH13 | 8 GH13 |
| | 3.2.1.3 | glucan 1,4-α-glucosidase | 207.2 | 178.2 | 65.63 | 7.03 | 291.5 | 367.8 | 2742 | 130.7 | 189.1 | 20 GH15 | 14 GH15 | 7 GH15 | 7 GH15 | 15 GH15 | 23 GH15 | 23 GH15 | 13 GH15 | 24 GH15 |
| | 3.2.1.4 | cellulase | 102.5 | 390.9 | 157.4 | 56.04 | 157.8 | 761.1 | 298.9 | 368.3 | 1263 | 1 GH12,25 GH5,4 GH9 | 1 GH12,26 GH5,14 GH9 | 1 GH12,21 GH5,1 GH9 | 1 GH12,2 GH5,5 GH9 | 1 GH12,15 GH5,7 GH9 | 2 GH12,1 GH44,3 GH5,3 GH9 | 4 GH12,2 GH44,9 GH5,7 GH9 | 1 GH12,2 GH44,6 GH5,8 GH9 | 1 GH12,1 GH44,3 GH5,9 GH9 |
| | 3.2.1.6 | endo-1,3(4)-β-glucanase | 179.6 | 424.3 | 476.4 | 8.710 | 809.0 | 255.7 | 193.5 | 1049 | 196.3 | 66 GH16 | 49 GH16 | 18 GH16 | 2 GH16 | 15 GH16 | 22 GH16 | 21 GH16 | 15 GH16 | 9 GH16 |
| | 3.2.1.8 | endo-1,4-β-xylanase | 110.5 | 760.4 | 984.9 | 73.20 | 1127 | 2757 | 5389 | 537.7 | 5928 | 44 GH10,4 GH11 | 16 GH10,12 GH11 | 24 GH10,12 GH11 | 5 GH10,1 GH11 | 29 GH10,4 GH11 | 7 GH10,3 GH11 | 36 GH10,5 GH11 | 10 GH10 | 11 GH10,3 GH11 |
| | 3.2.1.14 | chitinase | 245.9 | 441.8 | 368.7 | 2278 | 487.8 | 739.7 | 2059 | 493.5 | 540.0 | 31 GH18 | 24 GH18 | 14 GH18,1 GH19 | 31 GH18,2 GH19 | 16 GH18 | 42 GH18 | 46 GH18,2 GH19 | 24 GH18 | 42 GH18,1 GH19 |
| | 3.2.1.15 | polygalacturonase | 24.97 | 113.7 | 225.9 | 2.010 | 98.16 | 300.6 | 14.81 | 499.1 | 357.4 | 14 GH28 | 31 GH28 | 9 GH28 | 2 GH28 | 8 GH28 | 15 GH28 | 2 GH28 | 16 GH28 | 22 GH28 |
| | 3.2.1.17 | lysozyme | 8.970 | 3.600 | 2.160 | 79.68 | 3.860 | 11.82 | 82.62 | 94.42 | 10.22 | 10 GH25 | 1 GH25 | 1 GH25 | 4 GH22 | 1 GH25 | 4 GH25 | 3 GH22,3 GH25 | 1 GH25 | 4 GH25 |
| | 3.2.1.20 | α-glucosidase | 101.4 | 128.0 | 146.9 | 199.9 | 75.44 | 349.5 | 95.60 | 78.11 | 155.6 | 3 GH31 | 11 GH31 | 3 GH31 | 6 GH13,6 GH31,1 GH97 | 10 GH31 | 4 GH31 | 5 GH13,18 GH31 | 10 GH31 | 2 GH31 |
| | 3.2.1.21 | β-glucosidase | 565.3 | 333.9 | 536.1 | 267.0 | 910.5 | 960.7 | 7532 | 447.4 | 750.1 | 13 GH1,118 GH3 | 33 GH1,75 GH3 | 23 GH1,19 GH3 | 10 GH1,17 GH3 | 26 GH1,58 GH3 | 2 GH1,71 GH3 | 17 GH1,59 GH3 | 4 GH1,37 GH3 | 2 GH1,83 GH3 |
| | 3.2.1.22 | α-galactosidase | 9.870 | 143.7 | 173.1 | 26.20 | 283.2 | 281.2 | 43.42 | 80.90 | 192.4 | 7 GH27 | 20 GH27 | 13 GH27 | 6 GH27,1 GH36,1 GH4 | 17 GH27 | 8 GH27 | 10 GH27 | 3 GH27 | 3 GH27 |
| | 3.2.1.23 | β-galactosidase | 383.9 | 101.4 | 333.3 | 3.560 | 649.6 | 135.9 | 74.06 | 229.0 | 206.1 | 47 GH35 | 47 GH35 | 20 GH35 | 2 GH35 | 48 GH35 | 10 GH35 | 10 GH35 | 8 GH35 | 13 GH35 |
| | 3.2.1.24 | α-mannosidase | 58.46 | 54.62 | 75.00 | 131.9 | 104.5 | 92.92 | 76.59 | 42.30 | 77.48 | 8 GH38 | 3 GH38 | 5 GH38 | 5 GH38 | 5 GH38 | 2 GH38 | 8 GH38 | 10 GH38 | 4 GH38 |
| | 3.2.1.25 | β-mannosidase | 147.2 | 103.2 | 151.5 | 25.93 | 133.2 | 261.3 | 10.99 | 36.05 | 100.4 | 35 GH2 | 12 GH2 | 2 GH2 | 3 GH2 | 26 GH2 | 12 GH2 | 5 GH2 | 5 GH2 | 11 GH2 |

| | | | | | | | | | | | | | | | | | | | | | | |
|---|---|---|---|---|---|---|---|---|---|---|---|---|---|---|---|---|---|---|---|---|---|---|
| 3.2.1.28 | α,α-trehalase | 194.0 | 131.2 | 29.91 | 1976 | 225.4 | 216.8 | 96.59 | 100.9 | 463.6 | 7 GH37 | 13 GH37 | 4 GH37 | 21 GH37 | 7 GH37 | 2 GH37 | 6 GH37 | 8 GH37 | 3 GH37 |
| 3.2.1.31 | β-glucuronidase | 130.0 | 420.5 | 740.8 | 20.52 | 564.1 | 228.9 | 133.1 | 186.0 | 371.7 | 1 GH79 | 1 GH79 | 2 GH79 | 6 GH2 | 1 GH79 | 5 GH79 | 2 GH2,4 GH79 | 6 GH79 | 2 GH79 |
| 3.2.1.35 | hyaluronoglucosaminidase | - | - | - | 2.680 | - | 0.000 | 2.020 | - | - | - | - | - | 2 GH56 | - | - | 1 GH56 | - | - |
| 3.2.1.37 | xylan 1,4-β-xylosidase | 717.5 | 413.5 | 795.8 | 20.55 | 816.4 | 3742 | 252.9 | 317.9 | 317.9 | 7 GH3,4 GH5 | 22 GH3,6 GH5 | 5 GH3,6 GH5 | 4 GH3,3 GH43,5 GH5 | 29 GH3,4 GH5 | 6 GH3,3 GH5 | 5 GH3,3 GH5 | 5 GH3,3 GH5 | 9 GH3 |
| 3.2.1.39 | glucan endo-1,3-β-D-glucosidase | 312.9 | 307.1 | 385.2 | 1894 | 1032 | 635.7 | 2038 | 312.5 | 609.4 | 35 GH16,8 GH81 | 9 GH128,1 8 GH16,25 GH81 | 6 GH128,7 GH16,11 GH81 | 13 GH16 | 6 GH128,7 GH16,6 GH81 | 8 GH128,3 GH16,1 GH17,4 GH55,4 GH81 | 2 GH128,1 0 GH16,2 GH81 | 2 GH128,6 GH16,6 GH17,2 GH81 | 6 GH128,3 GH16,1 GH17,2 GH81 |
| 3.2.1.40 | α-L-rhamnosidase | 30.65 | 147.4 | 170.5 | 2.980 | 77.50 | 142.3 | 92.41 | 40.57 | 177.0 | 18 GH78 | 20 GH78 | 4 GH78 | 3 GH78 | 19 GH78 | 10 GH78 | 9 GH78 | 8 GH78 | 16 GH78 |
| 3.2.1.45 | glucosylceramidase | 85.66 | 67.19 | 51.70 | 6.010 | 70.82 | 214.3 | 53.72 | 53.67 | 179.5 | 11 GH5 | 5 GH5 | 5 GH5 | 5 GH30,1 GH5 | 6 GH5 | 16 GH5 | 1 GH30,11 GH5 | 9 GH5 | 6 GH5 |
| 3.2.1.50 | α-N-acetylglucosaminidase | - | - | - | 4.530 | - | - | 5.270 | - | - | - | - | - | 3 GH89 | - | - | 2 GH89 | - | - |
| 3.2.1.51 | α-L-fucosidase | - | 95.47 | 245.7 | 8.840 | 141.4 | 168.3 | 30.00 | - | 153.5 | 3 GH29 | 2 GH29 | 1 GH29 | 4 GH29 | 3 GH29 | 1 GH29 | 4 GH29 | - | 1 GH29 |
| 3.2.1.52 | β-N-acetylhexosaminidase | 50.71 | 305.4 | 159.5 | 54.11 | 222.5 | 301.2 | 310.8 | 206.4 | 514.1 | 7 GH20,2 GH3 | 7 GH20,7 GH3 | 12 GH20,9 GH3 | 17 GH20,1 GH3 | 4 GH20,16 GH3 | 6 GH20 | 17 GH20 | 5 GH20 | 10 GH20 |
| 3.2.1.55 | α-N-arabinofuranosidase | 33.76 | 44.47 | 352.4 | 6.250 | 218.9 | 1561 | 13.44 | 121.5 | 1594 | 44 GH51 | 2 GH51 | 1 GH51 | 1 GH43,1 GH51 | 23 GH51 | 2 GH51 | 7 GH51 | 2 GH51 | 4 GH51 |
| 3.2.1.58 | glucan 1,3-β-glucosidase | 1386.5 | 610.9 | 497.6 | 7.820 | 1213 | 955.4 | 414.3 | 464.3 | 674.8 | 27 GH5, | 39 GH5, | 15 GH5,7 GH55 | 4 GH5 | 14 GH5,9 GH55 | 13 GH5,15 GH55 | 23 GH5,18 GH55 | 9 GH5,6 GH55 | 23 GH5,4 GH55 |
| 3.2.1.59 | glucan endo-1,3-α-glucosidase | 64.61 | 30.15 | 41.77 | 22.33 | 77.36 | 13.77 | 25.21 | 28.69 | 25.00 | 5 GH71 | 4 GH71 | 7 GH71 | 6 GH87 | 6 GH71 | 2 GH71 | 1 GH71 | 3 GH71 | 4 GH71 |
| 3.2.1.63 | 1,2-α-L-fucosidase | 155.4 | 49.22 | 23.07 | 2.650 | 162.7 | 37.66 | 46.30 | 17.84 | 58.12 | 1 GH95 | 3 GH95 | 4 GH95 | 2 GH95 | 1 GH95 | 1 GH95 | 2 GH95 | 2 GH95 | 1 GH95 |
| 3.2.1.67 | galacturan 1,4-α-galacturonidase | 223.9 | 26.45 | 123.7 | 0.000 | 98.29 | 302.8 | 17.38 | 49.92 | 194.4 | 8 GH28 | 5 GH28 | 2 GH28 | - | 8 GH28 | 1 GH28 | 6 GH28 | 1 GH28 | 1 GH28 |
| 3.2.1.68 | isoamylase | - | - | - | 0.840 | - | - | - | - | - | - | - | - | 1 GH13 | - | - | - | - | - |
| 3.2.1.75 | glucan endo-1,6-β-glucosidase | - | - | - | - | - | 4.910 | - | - | - | - | - | - | - | - | 1 GH30 | - | - | - |
| 3.2.1.76 | L-iduronidase | - | - | - | 1.320 | - | - | - | - | - | - | - | - | 1 GH39 | - | - | - | - | - |
| 3.2.1.78 | mannan endo-1,4-β-mannosidase | 37.92 | 42.51 | 34.01 | 5.780 | 260.9 | 60.05 | 9.170 | 22.14 | 119.9 | 1 GH5 | 3 GH5 | 1 GH5 | 2 GH5 | 1 GH5 | 6 GH5 | 4 GH5 | 8 GH5 | 2 GH5 |
| 3.2.1.89 | arabinogalactan endo-β-1,4-galactanase | 10.88 | 16.30 | 53.68 | 0.000 | 21.02 | 82.69 | 9.720 | 25.61 | 82.49 | 6 GH53 | 7 GH53 | 1 GH53 | - | 5 GH53 | 1 GH53 | 3 GH53 | 1 GH53 | 1 GH53 |
| 3.2.1.91 | cellulose 1,4-β-cellobiosidase (non-reducing end) | 51.11 | 121.6 | 212.3 | 4.350 | 473.7 | 88.54 | 577.7 | 20.10 | 230.9 | 18 GH6 | 11 GH6 | 7 GH6 | 1 GH6 | 7 GH6 | 13 GH6 | 4 GH6 | 5 GH6 | 9 GH6 |
| 3.2.1.96 | mannosyl-glycoprotein endo-β-N-acetylglucosaminidase | - | - | - | 2.970 | - | 3.550 | - | - | - | - | - | - | 1 GH85 | - | 1 GH18 | - | - | - |
| 3.2.1.99 | arabinan endo-1,5-α-L-arabinanase | 28.14 | 47.01 | 102.1 | - | 83.13 | 86.58 | 16.27 | 81.97 | 362.3 | 8 GH43 | 1 GH43 | 1 GH43 | - | 16 GH43 | 1 GH43 | 10 GH43 | 2 GH43 | 1 GH43 |
| 3.2.1.106 | mannosyl-oligosaccharide glucosidase | 25.13 | 38.49 | 33.28 | 2.990 | 53.45 | 47.97 | 25.10 | 20.10 | 39.80 | 10 GH63 | 14 GH63 | 8 GH63 | 1 GH63 | 1 GH63 | 2 GH63 | 3 GH63 | 4 GH63 | 2 GH63 |
| 3.2.1.113 | mannosyl-oligosaccharide 1,2-α-mannosidase | 275.8 | 397.2 | 397.2 | 20.52 | 776.2 | 2671 | 539.7 | 825.6 | 1399 | 41 GH47 | 47 GH47 | 57 GH47 | 12 GH47,1 GH92 | 38 GH47 | 23 GH47 | 22 GH47 | 16 GH47 | 25 GH47 |
| 3.2.1.114 | mannosyl-oligosaccharide 1,3-1,6-α- | - | - | - | 4.200 | - | - | 7.650 | - | - | - | - | - | 4 GH38 | - | - | 2 GH38 | - | - |

| | | | | | | | | | | | | | | | | | | | |
|---|---|---|---|---|---|---|---|---|---|---|---|---|---|---|---|---|---|---|---|
| | mannosidase | | | | | | | | | | | | | | | | | | |
| 3.2.1.130 | glycoprotein endo-α-1,2-mannosidase | - | - | - | 0.870 | - | - | 1.210 | - | - | - | - | - | 1 GH99 | - | - | 1 GH99 | - | - |
| 3.2.1.131 | xylan α-1,2-glucuronosidase | 41.80 | 140.5 | 150.8 | - | 134.6 | 134.6 | 18.49 | 17.21 | 177.0 | 12 GH115 | 6 GH115 | 3 GH115 | - | 4 GH115 | 3 GH115,1 GH67 | 6 GH115 | 2 GH115 | 1 GH115 |
| 3.2.1.132 | chitosanase | - | - | - | - | - | 13.54 | 11.08 | 14.43 | 17.89 | - | - | - | - | - | 11 GH46 | 6 GH46 | 2 GH46 | 4 GH46 |
| 3.2.1.145 | galactan 1,3-β-galactosidase | 37.09 | 42.69 | 35.22 | 1.830 | 22.96 | 89.03 | 14.68 | 19.14 | 86.73 | 26 GH43 | 16 GH43 | 7 GH43 | 5 GH43 | 6 GH43 | 2 GH43 | 11 GH43 | 12 GH43 | 2 GH43 |
| 3.2.1.151 | xyloglucan-specific endo-β-1,4-glucanase | 36.04 | 112.6 | 104.9 | 0.50 | 720.6 | 100.7 | 100.4 | 90.91 | 167.1 | 4 GH12,7 GH74 | 4 GH12,13 GH74 | 2 GH12,5 GH74 | 2 GH74 | 2 GH12,6 GH74 | 2 GH12,9 GH74 | 4 GH12,20 GH74 | 2 GH12,7 GH74 | 2 GH12,5 GH74 |
| 3.2.1.166 | heparanase | - | - | - | 3.130 | - | - | 2.150 | - | - | - | - | - | 2 GH79 | - | - | 1 GH79 | - | - |
| 3.2.1.169 | protein O-GlcNAcase | - | - | - | 3.610 | - | - | 0.980 | - | - | - | - | - | 2 GH84 | - | - | 1 GH84 | - | - |
| 3.2.1.174 | rhamnogalacturonan rhamnohydrolase | 94.06 | 15.68 | 10.02 | - | 24.36 | 116.4 | 9.770 | 44.10 | 940.7 | 19 GH28 | 15 GH28 | 2 GH28 | - | 6 GH28 | 3 GH28 | 4 GH28 | 3 GH28 | 1 GH28 |
| 3.2.1.176 | cellulose 1,4-β-cellobiosidase (reducing end) | 41.81 | 310.5 | 1398 | 115.3 | 437.5 | 17403 | 7575 | 93.40 | 26124 | GH7 | GH7 | GH7 | GH7 | GH7 | GH7 | GH7 | GH7 | GH7 |
| 3.2.1.177 | α-D-xyloside xylohydrolase | 53.50 | 129.3 | 128.3 | 1.790 | 119.8 | 60.71 | 26.47 | 33.72 | 59.43 | 51 GH31 | 30 GH31 | 17 GH31 | 1 GH31 | 34 GH31 | 13 GH31 | 10 GH31 | 4 GH31 | 11 GH31 |
| 3.2.1.185 | β-L-arabinofuranosidase (non-reducing) | - | - | - | 0.820 | - | - | - | - | - | - | - | - | 1 GH127 | - | - | - | - | - |
| | Sum | **6 705** | **7 880** | **10 510** | **7 439** | **13 675** | **37 016** | **31 214** | **7 483** | **45 298** | **944** | **825** | **466** | **294** | **649** | **470** | **585** | **371** | **449** |
| | Total sum | **10 720** | **21 364** | **29 340** | **88 009** | **43 071** | **44 823** | **39 957** | **27 479** | **56 558** | **1320** | **1 090** | **612** | **373** | **906** | **664** | **850** | **546** | **692** |

Table S5. Predicted enzyme families (CAZymes) and expression level of transcript sequences represented in the *Termitomyces* transcriptomes in different sites of the decomposition process in *Macrotermes natalensis* and *Odontotermes* sp. The numbers refer to the number of transcript sequences identified and their expression levels (in Transcript per Million). White represents the lowest number of transcripts, yellow intermediate and red highest number. NA: we were not able to calculate the expression level.

| Predicted substrate | EC # | Predicted function | Expression level (Transcript per Million) | | | | | | | | | # of transcript sequences placed in different CAZy families | | | | | | | | |
|---|---|---|---|---|---|---|---|---|---|---|---|---|---|---|---|---|---|---|---|---|
| | | | *Macrotermes natalensis* 156 | | | *Odontotermes* sp. | | | | | | *Macrotermes natalensis* 156 | | | *Odontotermes* sp. | | | | | |
| | | | Nodules | Fresh comb | Old comb | Nodules (Od127) | Fresh comb (Od127) | Old comb (127) | Nodules (Od128) | Fresh comb (Od128) | Old comb (Od128) | Nodules | Fresh comb | Old comb | Nodules (Od127) | Fresh comb (Od127) | Old comb (127) | Nodules (Od128) | Fresh comb (Od128) | Old comb (Od128) |
| **Cellulose** | LPMO | AA9 expanded family | 600.8 | 197.5 | 177.3 | 27.12 | 677.3 | 580.3 | 161.6 | 341.7 | 2 257 | 21 AA9 | 18 AA9 | 16 AA9 | NA | NA | NA | NA | NA | NA |
| | 1.1.99.18 | Cellobiose dehydrogenase (acceptor) | 196.0 | 453.2 | 302.1 | 46.79 | 635.7 | 266.6 | 166.1 | 84.80 | 96.73 | 12 AA3,3 AA8 | 7 AA3, 3 AA8 | 5 AA3,3 AA8 | 8 AA3 | 7 AA3, 1 AA8 | 12 AA3, 1 AA8 | 19 AA3, 2 AA8 | 7 AA3,1 AA8 | 16 AA3, 2 AA8 |
| | 1.1.99.29 | Pyranose dehydrogenase (acceptor) | 13.77 | 36.08 | 8.600 | - | 30.27 | 7.200 | 13.88 | 27.29 | 4.790 | 4 AA3 | 18 AA3 | 4 AA3 | - | 8 AA3 | 3 AA3 | 2 AA3 | 8 AA3 | 1 AA3 |
| | 3.2.1.4 | Endo-1,4- β-D-glucanase | 102.5 | 176.3 | 181.9 | 56.04 | 796.2 | 1 059 | 393.1 | 562.0 | 1 526 | 1 GH12, 25 GH5, 4 GH9 | 1 GH12,2 6 GH5, 14 GH9 | 1 GH12, 21 GH5, 1 GH9 | 1 GH12, 2 GH5, 5 GH9 | 1 GH12, 15 GH5, 7 GH9 | 2 GH12, 1 GH44, 3 GH5, 3 GH9 | 4 GH12, 2 GH44, 9 GH5, 7 GH9 | 1 GH12, 2 GH44, 6 GH5, 8 GH9 | 1 GH12, 1 GH44, 3 GH5, 9 GH9 |
| | 3.2.1.21 | β-glucosidase | 565.3 | 333.9 | 536.1 | 267.0 | 910.5 | 960.7 | 7 532 | 447.4 | 750.1 | 13 GH1, 118 GH3 | 33 GH1, 75 GH3 | 23 GH1,19 GH3 | 10 GH1, 17 GH3 | 26 GH1, 58 GH3 | 2 GH1, 71 GH3 | 17 GH1, 59 GH3 | 4 GH1, 37 GH3 | 2 GH1, 83 GH3 |
| | 3.2.1.91 | Cellulose-1,4- β-cellobiosidase (non-reducing end) | 51.11 | 121.6 | 212.3 | 4.350 | 473.7 | 88.54 | 577.7 | 81.97 | 230.9 | 18 GH6 | 11 GH6 | 7 GH6 | 1 GH6 | 7 GH6 | 13 GH6 | 4 GH6 | 5 GH6 | 9 GH6 |
| | 3.2.1.176 | Cellulose-1,4- β-cellobiosidase (reducing end) | 41.81 | 310.5 | 1 398 | 115.3 | 437.5 | 17 403 | 7 575 | 93.40 | 26 124 | 9 GH7 | 11 GH7 | 5 GH7 | 3 GH7 | 8 GH7 | 9 GH7 | 11 GH7 | 6 GH7 | 8 GH7 |
| | | Sum | **1 571** | **1 629** | **2 816** | **517** | **3 961** | **20 365** | **16 420** | **1 639** | **30 990** | **228** | **217** | **105** | **47** | **138** | **120** | **136** | **85** | **135** |
| **Hemi-cellulose** | 3.1.1.6 | acetylesterase | 30.75 | 121.5 | 251.1 | 0 | 122.3 | 40.95 | 253.7 | 41.65 | 45.73 | 5 CE16 | 4 CE16 | 3 CE16 | 0 | 3 CE16 | 3 CE16 | 4 CE16 | 5 CE16 | 3 CE16 |
| | 3.1.1.73 | Feruloyl esterase | - | - | - | 1.100 | - | - | - | - | - | - | - | - | 3 CE1 | - | - | - | - | - |
| | 3.1.1.72 | Acetylxylan esterase | 142.4 | 235.1 | 488.1 | 4.450 | 799.2 | 230.3 | 217.1 | 145.3 | 775.7 | 6 CE1,10 CE4 | 4 CE1, 7 CE4 | 2 CE1, 4 CE4 | 2 CE1, 1 CE6 | 4 CE1, 11 CE4 | 3 CE1,6 CE4 | 2 CE1, 13 CE4 | 4 CE1, 5 CE4 | 5 CE1, 16 CE4 |
| | 3.2.1.55 | α-N-arabinofuranosidase | 33.76 | 44.47 | 352.4 | 6.250 | 218.9 | 1 561 | 13.44 | 121.5 | 1 594 | 44 GH51 | 2 GH51 | 1 GH51 | 1 GH43, 1 GH51 | 23 GH51 | 2 GH51 | 7 GH51 | 2 GH51 | 4 GH51 |
| | 3.2.1.8 | Endo-1,4- β-xylanase | 110.5 | 760.4 | 984.9 | 73.20 | 1 127 | 2 757 | 5 389 | 537.7 | 5 928 | 44 GH10, 4 GH11 | 16 GH10, 12 GH11 | 24 GH10, 12 GH11 | 5 GH10, 1 GH11 | 29 GH10, 4 GH11 | 7 GH10,3 GH11 | 36 GH10, 5 GH11 | 7 GH10, 3 GH11 | 11 GH10, 3 GH11 |
| | 3.2.1.37 | Xylan-1,4- β-xylosidase | 717.5 | 413.5 | 795.8 | 20.55 | 816.4 | 3 742 | 252.9 | 317.9 | 1976 | 7 GH3, 4 GH5 | 22 GH3, 6 GH5 | 5 GH3, 6 GH5 | 4 GH3, 3 GH43, 5 GH5 | 29 GH3, 4 GH5 | 6 GH3, 3 GH5 | 5 GH3, 3 GH5 | 5 GH3, 3 GH5 | 6 GH3, 3 GH5 |
| | 3.2.1.131 | Xylan-α-1,2-glucuronosidase | 41.80 | 140.5 | 150.8 | - | 134.6 | 114.0 | 18.49 | 17.21 | 177.0 | 12 GH115 | GH115 | GH115 | - | 4 GH115 | 4 GH115 | 6 GH115 | 2 GH115 | 1 GH115 |
| | 3.2.1.177 | α-D-xyloside-xylohydrolase | 53.50 | 129.3 | 128.3 | 1.790 | 119.8 | 60.71 | 26.47 | 33.72 | 59.43 | 51 GH31 | GH31 | GH31 | 1 GH31 | 34 GH31 | 13 GH31, 1 GH67 | 10 GH31 | 4 GH31 | 11 GH31 |
| | 3.2.1.151 | xyloglucan-specific endo- β-1,4-glucanase | 36.04 | 112.6 | 104.9 | 0.500 | 720.6 | 100.7 | 100.4 | 90.91 | 167.1 | 4 GH12, 7 GH74 | 4 GH12, 13 GH74 | 2 GH12, 5 GH74 | 2 GH74 | 2 GH12, 6 GH74 | 2 GH12, 9 GH74 | 4 GH12, 20 GH74 | 2 GH12, 7 GH74 | 2 GH12, 5 GH74 |
| | | Sum | **1 166** | **1 957** | **3 256** | **107.8** | **4 059** | **8 605** | **6 272** | **1 306** | **10 723** | **198** | **126** | **84** | **29** | **153** | **61** | **115** | **49** | **70** |
| **Lignin** | 1.10.3.2 | Laccase | 550.4 | 1463 | 1917 | 63.29 | 2 536 | 748.0 | 1 637 | 4 606 | 478.3 | 134 AA1 | 86 AA1 | 45 AA1 | 19 AA1 | 69 AA1 | 61 AA1 | 78 AA1 | 43 AA1 | 69 AA1 |
| | 3.1.1.* | 4-O-methyl-glucuronoyl methylesterase | 47.97 | 346.6 | 809.8 | 2.42 | 100.6 | 1 040 | 48.57 | 112.2 | 942.6 | 10 CE15 | 5 CE15 | 28 CE15 | 1 CE15 | 12 CE15 | 1 CE15 | 3 CE15 | 6 CE15 | 4 CE15 |
| | 1.11.1.16 | Versatile peroxidase | 2.070 | 542.2 | 95.25 | - | 1 317 | 66.78 | 1.05 | 4.32 | 0.95 | 2 AA2 | 1 AA2 | 1 AA2 | - | 1 AA2 | 2 AA2 | 1 AA2 | 1 AA2 | 1 AA2 |

| | | | | | | | | | | | | | | | | | | | | |
|---|---|---|---|---|---|---|---|---|---|---|---|---|---|---|---|---|---|---|---|---|
| | | **Sum** | **600.4** | **2 352** | **2 822** | **65.71** | **3 953** | **1 855** | **1 686** | **4 722** | **1 422** | **146** | **92** | **74** | **20** | **82** | **64** | **82** | **50** | **74** |
| **Pectin** | 4.2.2.2 | Pectate lyase | 100.7 | 239.8 | 191.9 | 8.620 | 312.2 | 77.29 | 237.3 | 26.36 | 33.64 | 8 PL1, 19 PL3 | PL3 | 13 PL1, 9 PL3 | 1 PL1, 1 PL3 | 12 PL1, 9 PL3 | 12 PL1, 5 PL3 | 4 PL1, 5 PL3 | 1 PL1, 5 PL3 | 5 PL1, 6 PL3 |
| | 4.2.2.10 | Pectin lyase | - | - | - | - | - | - | - | 12.48 | - | - | - | - | - | - | - | - | PL1 | - |
| | 3.1.1.* | Pectin acetylesterase, rhamnogalacturonan acetylesterase | 65.90 | 67.51 | 175.4 | 3.530 | 157.4 | 275.4 | 106.8 | 133.8 | 340.5 | 17 CE12 | 10 CE12 | 5 CE12 | 1 CE12 | 30 CE12 | 5 CE12 | 12 CE12, 1 CE13 | 9 CE12 | 3 CE12 |
| | 3.1.1.11 | pectinesterase | 28.17 | 96.87 | 98.62 | 0.950 | 232.9 | 65.65 | 21.00 | 18.22 | 74.66 | 23 CE8 | 26 CE8 | 10 CE8 | 1 CE8 | 15 CE8 | 15 CE8 | 13 CE8 | 5 CE8 | 7 CE8 |
| | 3.2.1.15 | polygalacturonase | 24.97 | 113.7 | 225.9 | 2.010 | 98.16 | 342.7 | 14.81 | 499.1 | 190.3 | 14 GH28 | 31 GH28 | 9 GH28 | 2 GH28 | 8 GH28 | 15 GH28 | 2 GH28 | 16 GH28 | 22 GH28 |
| | 3.2.1.67 | Galacturan-1,4-α-galacturonidase | 223.9 | 26.45 | 123.7 | - | 98.29 | 302.8 | 17.38 | 49.92 | 194.4 | 8 GH28 | 5 GH28 | 2 GH28 | - | 8 GH28 | 1 GH28 | 6 GH28 | 1 GH28 | 1 GH28 |
| | 4.2.2.* | rhamnogalacturonan lyase | 57.96 | 58.57 | 84.37 | 5.910 | 126.0 | 90.03 | 42.93 | 10.02 | 49.58 | 18 PL4 | 12 PL4 | 4 PL4 | 4 PL4 | 31 PL4 | 2 PL4 | 8 PL4 | 10 PL4 | 6 PL4 |
| | 3.2.1.174 | Rhamnogalacturonan rhamnohydrolase | 94.06 | 15.68 | 10.02 | - | 24.36 | 116.4 | 9.77 | 44.1 | 940.7 | 19 GH28 | 15 GH28 | 2 GH28 | - | 6 GH28 | 3 GH28 | 4 GH28 | 3 GH28 | 1 GH28 |
| | 3.2.1.40 | α-L-rhamnosidase | 30.65 | 147.4 | 170.5 | 2.980 | 77.5 | 142.3 | 92.41 | 40.57 | 221.7 | 18 GH78 | 20 GH78 | 4 GH78 | 3 GH78 | 19 GH78 | 10 GH78 | 9 GH78 | 8 GH78 | 16 GH78 |
| | | **Sum** | **626.3** | **766.0** | **1 080** | **24.00** | **1127** | **1413** | **542.4** | **834.6** | **2 045** | **144** | **147** | **58** | **13** | **138** | **68** | **64** | **59** | **67** |
| **Starch** | 3.2.1.1 | α-amylase | 98.25 | 156.3 | 168 | 16.23 | 132.0 | - | 26.01 | 14.71 | 13.94 | 15 GH13 | 12 GH13 | 3 GH13 | 3 GH13,1 GH57 | 15 GH13 | - | 4 GH13 | 5 GH13 | 8 GH13 |
| | 3.2.1.3 | Glucan-1,4-α-glucosidase | 207.2 | 178.2 | 65.63 | 7.030 | 291.5 | 367.8 | 2 742 | 130.7 | 189.1 | 20 GH15 | 14 GH15 | 7 GH15 | 7 GH15 | 15 GH15 | 23 GH15 | 23 GH15 | 13 GH15 | 24 GH15 |
| | 3.2.1.20 | α-glucosidase | 101.4 | 128.0 | 146.9 | 199.9 | 75.44 | 349.5 | 95.6 | 78.11 | 155.6 | 3 GH31 | 11 GH31 | 3 GH31 | 6 GH13, 6 GH31, 1 GH97 | 10 GH31 | 4 GH31 | 5 GH13, 18 GH31 | 10 GH31 | 2 GH31 |
| | 3.2.1.68 | isoamylase | - | - | - | 0.840 | - | - | - | - | - | - | - | - | 1 GH13 | - | - | - | - | - |
| | | **Sum** | **406.9** | **462.5** | **380.2** | **224.0** | **499.0** | **717.2** | **2 864** | **223.5** | **358.7** | **38** | **37** | **13** | **25** | **40** | **27** | **50** | **28** | **34** |
| **Arabinogalactan** | 3.2.1.89 | Arabinogalactan endo-β-1,4-galactanase | 10.88 | 16.30 | 53.68 | - | 21.02 | 82.69 | 9.720 | 25.61 | 82.49 | 6 GH53 | 7 GH53 | 1 GH53 | - | 5 GH53 | 1 GH53 | 3 GH53 | 1 GH53 | 1 GH53 |
| | 3.2.1.22 | α-galactosidase | 9.870 | 143.7 | 173.1 | 26.20 | 283.2 | 281.2 | 43.42 | 80.90 | 192.4 | 7 GH27 | 20 GH27 | 13 GH27 | 6 GH27, 1 GH36, 1 GH4 | 17 GH27 | 8 GH27 | 10 GH27 | 3 GH27 | 3 GH27 |
| | 3.2.1.23 | β-galactosidase | 383.9 | 101.4 | 348.8 | 3.560 | 649.6 | 135.9 | 74.06 | 229.0 | 206.1 | 47 GH35 | 47 GH35 | 20 GH35 | 2 GH35 | 48 GH35 | 10 GH35 | 10 GH35 | 8 GH35 | 13 GH35 |
| | 3.2.1.99 | Arabinan endo-1,5-α-L-arabinanase | 28.14 | 47.01 | 102.1 | - | 83.13 | 86.58 | 16.27 | 24.38 | 362.3 | 8 GH43 | 1 GH43 | 1 GH43 | - | 16 GH43 | 1 GH43 | 10 GH43 | 2 GH43 | 1 GH43 |
| | 3.2.1.145 | Galactan 1,3-β-galactosidase | 37.09 | 42.69 | 35.22 | 1.830 | 22.96 | 89.03 | 14.68 | 19.14 | 86.73 | 26 GH43 | 16 GH43 | 7 GH43 | 5 GH43 | 6 GH43 | 2 GH43 | 11 GH43 | 12 GH43 | 2 GH43 |
| | | **Sum** | **469.9** | **351.1** | **713.0** | **31.59** | **1060** | **675.4** | **158.2** | **379.0** | **930.0** | **94** | **91** | **42** | **15** | **92** | **22** | **44** | **26** | **20** |
| **Plant mannan** | 3.2.1.78 | Mannan endo-1,4-β-mannosidase | 37.92 | 42.51 | 34.01 | 5.780 | 260.9 | 60.05 | 9.170 | 22.14 | 119.9 | 1 GH5 | 3 GH5 | 1 GH5 | 2 GH5 | 1 GH5 | 6 GH5 | 4 GH5 | 8 GH5 | 2 GH5 |
| | 3.2.1.25 | β -mannosidase | 147.2 | 103.2 | 151.5 | 25.93 | 133.2 | 261.3 | 10.99 | 36.05 | 100.4 | 35 GH2 | 12 GH2 | 2 GH2 | 3 GH2 | 26 GH2 | 8 GH2 | 5 GH2 | 5 GH2 | 11 GH2 |
| | Sum | | **185.1** | **145.7** | **185.5** | **31.70** | **394.1** | **321.4** | **20.20** | **58.20** | **220.3** | **36** | **15** | **3** | **5** | **27** | **14** | **9** | **13** | **13** |
| | Total Sum | | **5 026** | **7 663** | **11 253** | **1 001** | **15 053** | **33 952** | **27 962** | **9 161** | **46 688** | **884** | **725** | **379** | **154** | **670** | **376** | **500** | **310** | **413** |

Table S6: Expression level (Transcript per Million) and distribution of transcripts (number of reads) across different sites of the decomposition process in one *Macrotermes natalensis* colony and two *Odontotermes* sp. colonies. Functions were predicted using Hotpep (PPR), sorted by substrate targets and listed according to predicted functions based on EC number. White represents the lowest number of transcripts, yellow intermediate and red highest number. NA: We were not able to place the transcripts within CAZy families.

| Termite species | Colony code | Sample | AZCL substrates enzyme activities ($cm^2$) | | | | | | | | | | | | | | | | |
|---|---|---|---|---|---|---|---|---|---|---|---|---|---|---|---|---|---|---|---|
| | | | **Amylose** | **Casein** | **Collagen** | **Debranched Arabinan** | **Rhamnogalacturonan** | **Galactomannan** | **Galactan** | **HE-Cellulose** | **Barley β-Glucan** | **Xyloglucan** | **Pachyman** | **Curdlan** | **Xylan** | **Arabinoxylan** | **Dextran** | **Pullulan** | **Chitosan** |
| *O.* sp. | Od127 (2015-2016) | Nodules | - | 0.12 | - | - | - | - | - | 2.20 | 2.54 | - | - | - | 2.94 | 2.24 | - | - | - |
| *O.* sp. | Od128 | Nodules | - | 0.76 | - | - | - | - | - | 3.17 | 3.65 | - | - | - | 3.43 | 4.10 | - | - | - |
| *O.* cf. *badius* | Od145 | Nodules | - | 1.02 | 0.44 | - | - | 0.95 | 0.34 | 3.00 | 3.00 | - | - | - | 5.05 | 3.44 | - | - | - |
| *O.* cf. *badius* | Od150 | Nodules | - | 0.65 | 0.85 | - | - | - | - | 2.27 | 2.09 | - | - | - | 4.87 | 4.75 | - | - | - |
| *O.* cf. *badius* | Od151 | Nodules | - | - | - | - | - | - | - | 1.59 | 2.12 | - | - | - | 3.71 | 4.96 | - | - | - |
| *O.* sp. | Od159 | Nodules | - | - | - | - | - | - | 0.47 | 2.40 | 1.96 | - | - | - | 3.55 | 3.54 | - | - | - |
| *M. natalensis* | Mn156 | Nodules | - | 0.87 | 0.82 | 0.68 | - | 2.39 | 0.42 | 5.02 | - | 1.83 | - | - | 5.03 | 3.95 | - | - | - |
| *M. natalensis* | Mn160 | Nodules | - | 1.11 | 1.81 | 0.89 | 0.45 | 2.33 | 0.80 | 4.62 | 5.02 | 2.52 | - | - | 6.72 | 5.72 | - | - | - |
| *M. natalensis* | Mn162 | Nodules | - | 1.26 | 0.75 | 2.49 | 1.07 | 2.45 | 1.28 | 5.16 | 4.12 | 3.29 | - | - | 6.56 | 8.01 | - | - | - |
| *M. natalensis* | Mn154 | Nodules | - | 1.83 | 1.99 | 0.92 | 0.94 | 2.04 | 1.42 | 4.27 | 4.06 | 2.86 | - | - | 4.47 | 4.22 | - | - | - |
| *M. natalensis* | Mn173 | Nodules | - | - | - | - | - | - | - | 1.65 | 2.14 | - | - | - | 1.15 | - | - | - | - |
| *M. natalensis* | Mn164 | Nodules | - | 1.36 | 1.19 | 1.75 | 1.33 | 2.08 | 1.65 | 4.53 | 4.35 | 1.24 | - | - | 5.96 | 5.25 | - | - | - |
| *O.* sp. | Od127 (2015-2016) | O. mj. w. guts | 1.74 | 0.50 | - | - | - | - | - | 1.58 | 2,46 | - | - | - | 1.73 | 1.82 | - | - | - |
| *O.* sp. | Od128 | O. mj. w. guts | 1.81 | 2.05 | 1.77 | 1.06 | - | - | - | 2.07 | 4.26 | - | - | - | 2.44 | 1.66 | - | - | - |
| *O.* cf. *badius* | Od145 | O. mj. w. guts | 1.92 | 0.57 | - | 0.40 | - | 0.55 | 0.45 | 2.25 | 3.93 | 0.33 | - | - | 1.14 | 1.74 | - | - | - |
| *O.* cf. *badius* | Od150 | O. mj. w. guts | 1.73 | 0.87 | 0.66 | - | - | - | - | 2.16 | 3.32 | - | - | - | 1.54 | 2.52 | - | - | - |
| *O.* cf. *badius* | Od151 | O. mj. w. guts | 1.85 | 0.73 | - | - | - | 0.49 | - | 1.80 | 3.16 | - | - | - | 1.92 | 2.50 | - | - | - |
| *O.* sp. | Od159 | O. mj. w. guts | 1.77 | 1.30 | 0.72 | - | - | - | - | 2.12 | 3.52 | - | - | - | 1.56 | 2.06 | - | - | - |
| *M. natalensis* | Mn156 | O. mj. w. guts | 1.63 | 1.21 | 0.98 | - | - | 0.91 | 0.92 | 2.51 | 4.75 | 0.70 | - | 0.33 | 3.79 | 4.83 | - | - | - |
| *M. natalensis* | Mn160 | O. mj. w. guts | 1.62 | 2.43 | 1.59 | 1.22 | 0.95 | 1.74 | 2.05 | 3.95 | 5.06 | 0.80 | - | 0.79 | 5.28 | 4.22 | - | - | - |
| *M. natalensis* | Mn162 | O. mj. w. guts | 1.71 | 0.80 | - | - | - | 0.28 | - | 2.16 | 4.84 | 0.24 | - | 0.59 | 4.01 | 4.72 | - | - | - |
| *M. natalensis* | Mn154 | O. mj. w. guts | 2.06 | 1.57 | 1.58 | - | - | 1.39 | 1.66 | 2.48 | 2.91 | 1.48 | - | 0.55 | 3.50 | 3.90 | - | - | - |
| *M. natalensis* | Mn173 | O. mj. w. guts | 1.82 | 1.44 | 1.70 | 0.99 | - | 1.23 | 1.09 | 3.09 | 3.08 | 0.96 | - | 0.56 | 3.27 | 2.96 | - | - | - |
| *M. natalensis* | Mn164 | O. mj. w. guts | 2.09 | 1.82 | 1.57 | - | - | 1.37 | 1.38 | 3.02 | 3.75 | 1.16 | - | 0.55 | 3.40 | 2.88 | - | - | - |
| *O.* sp. | Od127 (2016) | Y. mj. w. guts | 1.51 | 1.32 | - | - | - | - | - | 0.92 | 1.43 | - | - | - | 0.81 | 0.77 | - | - | - |
| *M. natalensis* | Mn154 | Y. mj. w. guts | 1.90 | 1.54 | 1.06 | - | - | 1.02 | 1.21 | 2.48 | 2.90 | 1.00 | - | 0.75 | 2.43 | 3.28 | - | - | - |
| *M. natalensis* | Mn173 | Y. mj. w. guts | 1.44 | 1.47 | 1.36 | 0.93 | - | 1.56 | 1.41 | 2.68 | 3.34 | 1.00 | - | 0.79 | 3.06 | 3.13 | - | - | - |
| *M. natalensis* | Mn164 | Y. mj. w. guts | 2.10 | 1.76 | 2.54 | - | - | 0.99 | 1.12 | 2.91 | 3.00 | 1.45 | - | 0.72 | 3.01 | 2.95 | - | - | - |
| *O.* sp. | Od127 (2016) | O. mi. w. guts | 1.68 | 1.37 | - | - | - | - | - | 2.09 | 2.54 | - | - | - | 2.33 | 2.60 | - | - | - |
| *M. natalensis* | Mn154 | O. mi. w. guts | 1.69 | 1.64 | 1.81 | - | - | 1.57 | 1.41 | 2.58 | 2.81 | 1.58 | - | 0.59 | 2.66 | 3.52 | - | - | - |
| *M. natalensis* | Mn173 | O. mi. w. guts | 1.73 | 1.77 | 1.25 | 1.01 | - | 1.56 | 1.21 | 2.30 | 3.06 | 0.93 | - | 0.55 | 3.49 | 3.35 | - | - | - |
| *M. natalensis* | Mn164 | O. mi. w. guts | 1.83 | 1.99 | 2.46 | - | - | 1.59 | 1.22 | 2.75 | 3.72 | 2.40 | - | 0.62 | 3.23 | 3.42 | - | - | - |
| *O.* sp. | Od127 (2016) | O. mi. w. guts | 1.51 | 1.10 | - | - | - | - | - | 0.74 | 1.29 | - | - | - | 0.75 | 0.60 | - | - | - |
| *M. natalensis* | Mn154 | O. mi. w. guts | 1.62 | 1.72 | 1.64 | - | - | 1.47 | 1.33 | 2.71 | 2.65 | 1.11 | - | 0.79 | 2.71 | 3.06 | - | - | - |
| *M. natalensis* | Mn173 | O. mi. w. guts | 1.33 | 1.61 | 1.46 | 0.76 | - | 1.40 | 1.14 | 2.45 | 3.01 | 1.15 | - | 0.79 | 2.91 | 3.43 | - | - | - |
| *M. natalensis* | Mn164 | O. mi. w. guts | 3.15 | 1.51 | 2.10 | - | - | 1.57 | 1.11 | 2.89 | 3.22 | 1.14 | - | 0.79 | 3.65 | 3.49 | - | - | - |
| *O.* sp. | Od127 (2015-2016) | Fresh comb | - | - | - | - | - | - | - | 0.98 | 2.16 | - | - | - | 1.29 | 1.15 | - | - | - |
| *O.* sp. | Od128 | Fresh comb | - | - | - | - | - | - | - | 0.46 | 1.67 | - | - | - | 2.78 | 3.30 | - | - | - |
| *O.* cf. *badius* | Od145 | Fresh comb | - | - | - | - | - | - | - | - | 1.33 | - | - | - | 2.05 | 1.17 | - | - | - |
| *O.* cf. *badius* | Od150 | Fresh comb | - | - | - | - | - | - | - | 1.87 | 2.25 | - | - | - | 3.56 | 2.35 | - | - | - |
| *O.* cf. *badius* | Od151 | Fresh comb | - | - | - | - | - | - | - | 3.14 | 3.61 | - | - | - | 2.92 | 1.87 | - | - | - |
| *O.* sp. | Od159 | Fresh comb | - | - | - | - | - | - | - | 1.68 | 0.94 | - | - | - | 2.35 | 0.84 | - | - | - |
| *M. natalensis* | Mn156 | Fresh comb | - | - | - | - | - | - | - | 0.25 | 0.70 | - | - | - | 0.31 | 0.23 | - | - | - |
| *M. natalensis* | Mn160 | Fresh comb | - | - | - | 0.49 | - | - | - | 2.01 | 3.42 | 0.50 | - | - | 1.83 | 2.16 | - | - | - |
| *M. natalensis* | Mn162 | Fresh comb | - | - | - | 0.56 | - | - | - | 1.70 | 1.61 | - | - | - | 1.95 | 1.62 | - | - | - |
| *M. natalensis* | Mn154 | Fresh comb | - | - | - | - | - | - | - | 1.88 | 2.03 | - | - | - | 1.83 | 1.30 | - | - | - |
| *M. natalensis* | Mn173 | Fresh comb | - | - | - | - | - | 1.13 | - | 2.14 | 2.05 | 1.12 | - | - | 3.10 | 2.91 | - | - | - |
| *M. natalensis* | Mn164 | Fresh comb | - | - | - | - | - | - | - | 1.75 | 2.10 | - | - | - | 1.29 | 1.51 | - | - | - |
| *O.* sp. | Od127 (2015-2015) | Old comb | - | - | - | - | - | - | - | 0.94 | 2.02 | - | - | - | 2.09 | 1.72 | - | - | - |
| *O.* sp. | Od128 | Old comb | - | - | - | - | - | - | - | 1.24 | 2.73 | - | - | - | 1.96 | 3.02 | - | - | - |
| *O.* cf. *badius* | Od145 | Old comb | - | - | - | - | - | - | - | 1.50 | 1.99 | 0.43 | - | - | 2.12 | 1.51 | - | - | - |
| *O.* cf. *badius* | Od150 | Old comb | - | - | - | - | - | - | - | 2.50 | 2.56 | - | - | - | 3.38 | 3.11 | - | - | - |
| *O.* cf. *badius* | Od151 | Old comb | - | - | - | - | - | - | - | 1.49 | 0.78 | - | - | - | 0.71 | 0.11 | - | - | - |
| *O.* sp. | Od159 | Old comb | - | - | - | - | - | - | - | 1.53 | 1.30 | - | - | - | 2.69 | 1.48 | - | - | - |
| *M. natalensis* | Mn156 | Old comb | - | - | - | - | - | - | - | 0.68 | 2.16 | - | - | - | 1.05 | 0.95 | - | - | - |
| *M. natalensis* | Mn160 | Old comb | - | - | - | - | - | - | - | - | - | - | - | - | 1.15 | 0.74 | - | - | - |
| *M. natalensis* | Mn162 | Old comb | - | - | - | - | - | - | - | - | - | - | - | - | 0.0 | 0.14 | - | - | - |
| *M. natalensis* | Mn154 | Old comb | - | - | - | - | - | - | - | - | 0.98 | - | - | - | 1.48 | 1.56 | - | - | - |
| *M. natalensis* | Mn173 | Old comb | - | - | - | - | - | - | - | 1.95 | 1.84 | 1.19 | - | - | 3.94 | 3.28 | - | - | - |
| *M. natalensis* | Mn164 | Old comb | - | - | - | - | - | - | - | - | 1.05 | - | - | - | 2.43 | 2.59 | - | - | - |

Table S7: AZCL enzyme activities in different samples collected in 2015 and 2016 after 24 hrs of incubation. The enzyme activities were measured as area ($cm^2$) of the halos produced by hydrolyses of AZCL substrates. *O.* sp: *Odontotermes* sp., *O.* cf. *badius: Odontotermes badius*, *M. natalensis*: *Macrotermes natalensis,* O. mj. w. guts: Old major worker guts, Y. mj. w. guts: Young major worker guts, O. mi. w. guts: Old minor worker guts.

| Termite species | Colony code | Sample | Chromogenic substrate enzyme activity (absorbance) | | | | | | | | | |
|---|---|---|---|---|---|---|---|---|---|---|---|---|
| | | | **Amylose** | **Rhamnogalacturonan** | **Galactomannan** | **HE-Cellulose** | **Barley β-Glucan** | **Xyloglucan** | **Pachyman** | **Curdlan** | **Xylan** | **Arabinoxylan** |
| | | | 595 nm | 517 nm | 595 nm | 595 nm | 595 nm | 595 nm | 595 nm | 595 nm | 595 nm | 595 nm |
| *M. natalensis* | Mn154 | Nodules | 0.049 | 1.279 | 2.259 | 3.689 | 3.468 | 3.232 | 0.033 | 1.043 | 3.687 | 3.007 |
| *M. natalensis* | Mn164 | Nodules | 0.048 | 1.267 | 2.220 | 3.827 | 3.535 | 2.903 | 0.100 | 1.237 | 3.726 | 3.644 |
| *M. natalensis* | Mn173 | Nodules | 0.027 | 0.092 | 0.282 | 2.094 | 2.003 | 0.121 | 1.977 | 0.681 | 3.725 | 0.045 |
| *M. natalensis* | Mn154 | O. mj. w. guts | 2.621 | 0.361 | 1.417 | 2.433 | 3.066 | 0.840 | 1.077 | 1.204 | 3.610 | 0.475 |
| *M. natalensis* | Mn164 | O. mj. w. guts | 2.378 | 0.279 | 0.915 | 3.734 | 3.059 | 0.453 | 1.147 | 1.173 | 3.731 | 0.307 |
| *M. natalensis* | Mn173 | O. mj. w. guts | 2.777 | 0.285 | 1.342 | 3.533 | 3.199 | 0.547 | 1.120 | 1.195 | 3.764 | 0.409 |
| *M. natalensis* | Mn154 | Y. mj. w. guts | 2.862 | 0.417 | 3.695 | 0.519 | 3.175 | 1.076 | 1.172 | 1.316 | 0.289 | 3.722 |
| *M. natalensis* | Mn164 | Y. mj. w. guts | 2.662 | 0.438 | 3.612 | 0.502 | 3.379 | 0.978 | 1.151 | 1.237 | 0.379 | 3.609 |
| *M. natalensis* | Mn173 | Y. mj. w. guts | 3.440 | 0.352 | 3.659 | 0.540 | 2.980 | 1.025 | 1.166 | 0.756 | 0.285 | 3.696 |
| *M. natalensis* | Mn154 | O. mi. w. guts | 1.892 | 0.334 | 3.748 | 0.464 | 3.170 | 1.180 | 1.188 | 1.182 | 0.241 | 3.763 |
| *M. natalensis* | Mn164 | O. mi. w. guts | 3.006 | 0.473 | 3.729 | 0.671 | 3.117 | 0.911 | 1.268 | 1.306 | 0.292 | 3.833 |
| *M. natalensis* | Mn173 | O. mi. w. guts | 2.588 | 0.298 | 3.690 | 0.693 | 3.426 | 1.016 | 1.118 | 1.398 | 0.288 | 3.691 |
| *M. natalensis* | Mn154 | Y. mi. w. guts | 2.837 | 0.594 | 3.686 | 0.789 | 3.151 | 1.115 | 1.178 | 1.107 | 0.503 | 3.694 |
| *M. natalensis* | Mn164 | Y. mi. w. guts | 2.307 | 0.630 | 3.709 | 0.663 | 3.301 | 1.168 | 1.122 | 1.511 | 0.500 | 3.731 |
| *M. natalensis* | Mn173 | Y. mi. w. guts | 2.723 | 0.397 | 3.739 | 0.564 | 3.210 | 1.318 | 1.265 | 0.889 | 0.402 | 3.750 |
| *M. natalensis* | Mn154 | Fresh comb | 0.284 | 0.237 | 0.551 | 2.509 | 2.770 | 0.331 | 0.009 | 0.218 | 3.084 | 0.240 |
| *M. natalensis* | Mn164 | Fresh comb | 0.074 | 0.145 | 0.123 | 1.185 | 1.133 | 0.453 | 0.013 | 0.266 | 2.696 | 0.170 |
| *M. natalensis* | Mn173 | Fresh comb | 0.002 | 0.657 | 1.629 | 3.515 | 3.474 | 1.018 | 0.013 | 0.491 | 3.621 | 1.213 |
| *M. natalensis* | Mn154 | Old comb | 0.048 | 0.750 | 1.014 | 3.108 | 1.205 | 0.533 | 0.058 | 1.163 | 3.621 | 0.929 |
| *M. natalensis* | Mn164 | Old comb | 0.021 | 0.262 | 0.115 | 0.703 | 0.773 | 0.664 | 0.018 | 0.649 | 3.245 | 0.264 |
| *M. natalensis* | Mn173 | Old comb | 0.033 | 0.683 | 0.880 | 1.675 | 0.936 | 0.519 | 0.035 | 1.207 | 3.667 | 0.678 |

Table S8: Enzyme activities in *Macrotermes natalensis* colony components detected after 24hr incubation of extracts with chromogenic polysaccharide hydrogel (CPH), expressed in absorbance measured at 595 and 517 nm for blue and red substrates, respectively. O. mj. w.: Old major worker, O. mj. w. guts: Old major worker guts, Y. mj. w. guts: Young major worker guts, O. mi. w. guts: Old minor worker guts, Y. mi..

| Termite species and sample | Glucosamine ratio old comb/fresh comb | Corrected fresh comb enzyme activities (cm²) | | | | | | | | |
|---|---|---|---|---|---|---|---|---|---|---|
| | | **Debranched Arabinan** | **Galactomannan** | **Galactan** | **HE-Cellulose** | **Barley β-Glucan** | **Xyloglucan** | **Pachyman** | **Xylan** | **Arabinoxylan** |
| *M. natalensis* Mn154 | 2.97 | 0.00 | 0.00 | 0.00 | 5.583 | 6.042 | 0.00 | 0.00 | 5.426 | 3.854 |
| *M. natalensis* Mn156 | 1.13 | 0.00 | 0.00 | 0.00 | 0.2778 | 0.7932 | 0.00 | 0.00 | 0.3500 | 0.2616 |
| *M. natalensis* Mn160 | 1.11 | 0.5495 | 0.00 | 0.00 | 2.240 | 3.806 | 0.5514 | 0.00 | 2.040 | 2.406 |
| *M. natalensis* Mn162 | 1.22 | 0.6843 | 0.00 | 0.00 | 2.077 | 1.975 | 0.00 | 0.00 | 2.3825 | 1.989 |
| *M. natalensis* Mn164 | 2.53 | 0.00 | 0.00 | 0.00 | 4.425 | 5.317 | 0.00 | 0.00 | 3.276 | 3.823 |
| *M. natalensis* Mn173 | 2.71 | 0.00 | 3.072 | 0.00 | 5.808 | 5.557 | 3.033 | 0.00 | 8.418 | 7.899 |
| *Odontotermes* sp. Od127 2015 | 1.29 | 0.00 | 0.00 | 0.00 | 1.506 | 3.923 | 0.00 | 0.00 | 2.332 | 2.021 |
| *Odontotermes* sp. Od127 2016 | 2.14 | 0.00 | 0.00 | 0.00 | 1.681 | 2.703 | 0.00 | 0.00 | 1.651 | 1.569 |
| *Odontotermes* sp. Od128 | 1.41 | 0.00 | 0.00 | 0.00 | 0.6527 | 2.353 | 0.00 | 0.7374 | 3.933 | 4.655 |
| *O.* cf. *badius* Od145 | 1.35 | 0.00 | 0.00 | 0.00 | 0.00 | 1.798 | 0.00 | 0.00 | 2.759 | 1.572 |
| *O.* cf. *badius* Od150 | 1.66 | 0.00 | 0.00 | 0.00 | 3.114 | 3.742 | 0.00 | 0.00 | 5.909 | 3.911 |
| *O.* cf. *badius* Od151 | 1.39 | 0.8070 | 0.00 | 1.980 | 4.345 | 5.001 | 0.00 | 0.00 | 4.047 | 2.584 |
| *Odontotermes* sp. Od159 | 1.23 | 0.00 | 0.00 | 0.00 | 2.057 | 1.148 | 0.00 | 0.00 | 2.875 | 1.026 |

Table S9: Normalization of AZCL enzyme activities in fresh comb samples based on the ratio of fungal biomass, measured as glucosamine (GlcN), in old comb/fresh comb.

| Sample | Lignin | Cellulose | Xylose | Glucose | Arabinose | Mannose | Galactose | Fucose | GalA | GlcA | TOTAL (%) |
|---|---|---|---|---|---|---|---|---|---|---|---|
| Mn154 FC | 11.23 | 11.15 | 9.382 | 7.019 | 2.491 | 0.7353 | 1.856 | 0.1121 | 0.4055 | 0.2835 | |
| Mn164 FC | 9.70 | 14.43 | 13.21 | 6.296 | 3.480 | 0.8155 | 2.368 | 0.09448 | 0.1480 | 0.2356 | |
| Mn173 FC | 12.25 | 15.62 | 13.83 | 6.477 | 3.280 | 1.079 | 2.045 | 0.1577 | 0.3463 | 0.3141 | **46.04** |
| Mn156 FC | 13.94 | 13.20 | 4.475 | 3.589 | 0.9845 | 0.5485 | 0.8291 | 0.1072 | 0.5992 | 0.2046 | |
| Mn160 FC | 14.44 | 16.01 | 3.844 | 3.529 | 1.027 | 0.6275 | 0.9554 | 0.1077 | 0.6979 | 0.2001 | |
| Mn162 FC | 12.29 | 13.98 | 11.63 | 3.457 | 2.373 | 0.4870 | 0.6714 | 0.1139 | 0.2733 | 0.1533 | |
| Od sp. 127 FC 2016 | 12.28 | 12.93 | 5.201 | 5.139 | 1.892 | 1.242 | 1.8999 | 0.1535 | 0.7220 | 0.3022 | |
| Od sp. 127 FC 2015 | 10.73 | 11.73 | 4.921 | 4.034 | 1.311 | 1.026 | 1.140 | 0.1742 | 0.7244 | 0.2432 | **38.57** |
| Od sp. 128 FC | 13.15 | 12.44 | 3.000 | 2.338 | 0.8705 | 0.7849 | 0.7112 | 0.07989 | 0.3342 | 0.2789 | |
| Od sp. 159 FC | 14.71 | 13.44 | 6.241 | 2.762 | 2.319 | 0.6689 | 1.0482 | 0.1291 | 0.8339 | 0.3293 | |
| Od badius 145 FC | 13.73 | 12.77 | 4.526 | 2.577 | 1.620 | 0.5936 | 1.065 | 0.1019 | 0.7825 | 0.3266 | |
| Od150 badius FC | 14.93 | 16.81 | 2.867 | 1.977 | 1.062 | 0.5236 | 0.5939 | 0.06880 | 0.6953 | 0.2378 | **38.59** |
| Od151 badius FC | 14.80 | 13.19 | 3.312 | 2.593 | 1.415 | 0.6341 | 0.8673 | 0.08664 | 0.6780 | 0.3095 | |
| Mn154 OC | 11.67 | 5.343 | 6.874 | 5.213 | 1.349 | 0.9080 | 1.3847 | 0.1095 | 0.4350 | 0.2394 | |
| Mn164 OC | 7.625 | 20.37 | 7.817 | 6.172 | 2.322 | 0.8106 | 1.7731 | 0.1073 | 0.1560 | 0.1929 | |
| Mn173 OC | 8.553 | 23.48 | 7.660 | 6.549 | 1.926 | 0.8130 | 1.3310 | 0.1363 | 0.1849 | 0.2000 | **44.87** |
| Mn156 OC | 13.86 | 14.42 | 9.236 | 3.589 | 0.9845 | 0.5485 | 0.8291 | 0.1072 | 0.5992 | 0.2046 | |
| Mn160 OC | 14.91 | 17.50 | 6.856 | 3.529 | 1.027 | 0.6275 | 0.9554 | 0.107 | 0.6979 | 0.2001 | |
| Mn162 OC | 13.70 | 17.63 | 7.823 | 3.457 | 2.373 | 0.4870 | 0.6714 | 0.1139 | 0.2733 | 0.1533 | |
| Od sp. 127 OC 2016 | 12.23 | 11.83 | 3.431 | 3.577 | 1.244 | 0.8900 | 1.2363 | 0.1168 | 0.4488 | 0.1904 | |
| Od sp. 127 OC 2015 | 8.020 | 8.782 | 4.921 | 4.034 | 1.311 | 1.026 | 1.140 | 0.1742 | 0.7244 | 0.2432 | **32.79** |
| Od sp. 128 OC | 14.46 | 11.45 | 3.217 | 3.546 | 0.9336 | 1.125 | 0.9484 | 0.0966 | 0.434434 | 0.1837 | |
| Od sp. 159 OC | 11.33 | 8.128 | 3.147 | 2.539 | 1.347 | 0.5937 | 1.003 | 0.09915 | 0.7432 | 0.2389 | |
| Od badius 145 OC | 14.06 | 10.28 | 4.425 | 2.863 | 1.616 | 0.6071 | 1.047 | 0.1055 | 0.8413 | 0.3320 | |
| Od badius 150 OC | 8.432 | 8.872 | 3.569 | 3.252 | 1.212 | 0.8281 | 0.7883 | 0.1269 | 1.196 | 0.2688 | **30.17** |
| Od badius 151 OC | 11.46 | 4.393 | 2.507 | 3.035 | 1.028 | 0.9050 | 0.8742 | 0.1290 | 1.193 | 0.2383 | |
| Mn154 Guts | 3.852 | 5.702 | 0.4493 | 5.262 | 0.2481 | 0.5871 | 0.5355 | 0.08002 | 0.05279 | 0.06171 | |
| Mn164 Guts | 3.387 | 0.9348 | 0.8548 | 5.100 | 0.4393 | 0.7185 | 0.6667 | 0.09170 | 0.08286 | 0.1317 | **14.44** |
| Mn173 Guts | 4.653 | 1.812 | 0.5539 | 5.102 | 0.3223 | 0.7483 | 0.6791 | 0.1052 | 0.03669 | 0.09004 | |
| Od127 Guts | 3.981 | 1.092 | 0.4245 | 4.112 | 0.2421 | 0.7122 | 0.6684 | 0.1245 | 0.05438 | 0.07543 | **11.48** |
| Od. sp Dry wood (F3) | 7.937 | 29.71 | 10.15 | 3.555 | 1.635 | 0.9685 | 1.6369 | 0.08277 | 2.548 | 0.4424 | |
| Od. sp Dry wood (F5) | 13.99 | 33.75 | 11.51 | 2.274 | 0.6169 | 0.3654 | 0.9148 | 0.03633 | 0.9965 | 0.1233 | **55.55** |
| Od. sp Dry wood (F6) | 12.48 | 35.76 | 12.89 | 4.035 | 0.2688 | 0.7064 | 0.9030 | 0.03540 | 1.015 | 0.1705 | |
| Od. sp Dry wood (F25) | 1.568 | 9.695 | 4.081 | 2.433 | 5.334 | 1.035 | 3.146 | 0.02495 | 3.097 | 0.2301 | |
| Od. badius Dry wood (F2) | 12.72 | 28.01 | 13.78 | 3.246 | 1.086 | 0.5878 | 1.203 | 0.05081 | 1.039 | 0.1436 | **52.89** |
| Od. badius Dry wood (F4) | 10.71 | 16.37 | 10.94 | 2.560 | 0.6439 | 0.6876 | 0.9684 | 0.07492 | 0.7777 | 0.1777 | |
| M. natalensis Dry wood (F11) | 15.37 | 31.20 | 1.862 | 4.173 | 0.2622 | 1.360 | 1.176 | 0 | 0.1293 | 0.1586 | |
| M. natalensis Dry wood (F16) | 16.23 | 36.85 | 8.597 | 1.933 | 0.8865 | 0.5701 | 0.9612 | 0.01620 | 0.7257 | 0.1222 | **64.76** |
| M. natalensis Dry wood (F17) | 14.45 | 23.54 | 14.06 | 4.474 | 1.030 | 1.246 | 1.677 | 0.0090 | 1.711 | 0.2516 | |
| M. natalensis Dry wood (F23) | 8.50 | 40.53 | 15.22 | 5.147 | 0.6424 | 1.435 | 1.294 | 0.01236 | 1.018 | 0.1606 | |
| O. sp. Cow dung (F15) | 13.93 | 26.10 | 5.912 | 1.633 | 1.336 | 0.3947 | 0.7156 | 0.007095 | 0.1303 | 0.1741 | **50.34** |
| O. badius Cow dung (F13) | 13.91 | 16.05 | 5.764 | 1.581 | 1.309 | 0.3014 | 0.7010 | 0.0060 | 0.1290 | 0.1632 | **39.92** |
| M. natalensis Cow Dung (F8) | 11.27 | 14.11 | 4.486 | 1.124 | 0.8899 | 0.1728 | 0.55885 | 0.005686 | 0.08768 | 0.09107 | **40.63** |
| M. natalensis Cow Dung (F14) | 13.30 | 26.08 | 3.741 | 1.954 | 1.806 | 0.3801 | 0.9033 | 0.005867 | 0.1082 | 0.175269 | |
| M. natalensis Decaying wood (F7) | 9.273 | 27.12 | 3.345 | 2.525 | 0.9487 | 0.5137 | 1.1652 | 0.03983 | 0.6215 | 0.1707 | **57.54** |
| M. natalensis Decaying wood (F22) | 20.61 | 28.19 | 13.12 | 4.454 | 0.3037 | 0.5174 | 0.959651 | 0.00768 | 1.069 | 0.1257 | |
| M. natalensis Bark(F9) | 14.86 | 35.90 | 3.055 | 3.797 | 0.0756 | 0.3025 | 0.297648 | 0 | 0.07216 | 0.1171 | **60.70** |
| M. natalensis Bark(F10) | 8.560 | 43.88 | 4.991 | 2.867 | 0.5584 | 0.3242 | 1.194028 | 0.003935 | 0.4262 | 0.0993 | |

Table S10: Content of polysaccharides expressed in % per gram of AIR sample, where cellulose content was measured by using 4% sulfuric acid hydrolysis, lignin was measured using Acetyl-bromide and non-cellulosic polymers were measured using trifluoroacetic acid.Mn: *Macrotermes natalensis,* Od sp.: *Odontotermes* sp., Od badius: *Odontotermes badius,* , GalA: Galacturonic acid, GlcA: Glucuronic acid.

| Treatment | Sample | (1->3) β-D-glucan (BS-400-2) | (1->3)(1->4) β-D-glucan (BS-400-3) | (1->4) β-D-mannan (BS-400-4) | Cellulose (CBM3a) | Extensin (LM1) | Arabinogalactan protein AGP (LM2) | (1->4) β-D-galactan (LM5) | (1->5) α-L-arabinan (DLM6) | Homogalacturonan with an intermediate DE (LM7) | Xylogalacturonan (LM8) | (1->4) β-D-xylan (LM10) | Xylan/Arabinoxylan (LM11) | Arabinan (LM13) | Xyloglucan (LM15) | Arabinan (LM16) | Homogalacturonan (LM18 (MUC2)) | Homogalacturonan (LM19 (XGA2)) | Homogalacturonan (LM20 (MUC1)) | Mannan (LM21) | Mannan LM22 | (1->4) β-D-xylan (LM23) | Xyloglucan (LM24) | Xyloglucan (LM25) | Homogalacturonan, low DE (JIM5) | No defined epitope, (Anti callose, MLG like binding) (JIM6) | Homogalacturonan, low DE (JIM7) |
|---|---|---|---|---|---|---|---|---|---|---|---|---|---|---|---|---|---|---|---|---|---|---|---|---|---|---|---|
| CDTA | *O.* sp.[1] 127 F.C. (2015-2016) | 3 | 0 | 0 | 3 | 0 | 4 | 0 | 0 | 0 | 0 | 0 | 0 | 0 | 0 | 0 | 3 | 3 | 0 | 0 | 2 | 0 | 0 | 1 | 2 | 18 | 6 |
| | *O.* sp. 128 F.C. | 3 | 0 | 0 | 2 | 0 | 5 | 0 | 0 | 0 | 0 | 0 | 0 | 0 | 0 | 0 | 4 | 4 | 0 | 0 | 2 | 5 | 0 | 3 | 2 | 20 | 7 |
| | *O.* sp. 159 F.C. | 0 | 0 | 0 | 3 | 0 | 2 | 0 | 0 | 0 | 0 | 0 | 0 | 0 | 0 | 0 | 5 | 4 | 0 | 0 | 2 | 0 | 0 | 2 | 3 | 14 | 7 |
| | *O.* cf. *badius* 145 F.C. | 1 | 0 | 0 | 2 | 1 | 3 | 0 | 0 | 0 | 0 | 0 | 0 | 0 | 0 | 0 | 4 | 4 | 0 | 0 | 1 | 0 | 0 | 3 | 3 | 16 | 7 |
| | *O.* cf. *badius* 150 F.C. | 3 | 0 | 0 | 3 | 2 | 4 | 0 | 1 | 0 | 2 | 0 | 0 | 0 | 0 | 0 | 3 | 4 | 0 | 0 | 3 | 0 | 0 | 3 | 2 | 17 | 7 |
| | *O.* cf. *badius* 151 F.C. | 0 | 0 | 0 | 5 | 0 | 7 | 0 | 0 | 0 | 0 | 0 | 0 | 2 | 0 | 0 | 0 | 0 | 0 | 0 | 4 | 0 | 0 | 3 | 0 | 12 | 4 |
| | Mn 156 F.C. | 2 | 0 | 0 | 3 | 0 | 3 | 0 | 0 | 0 | 0 | 0 | 0 | 0 | 0 | 0 | 7 | 5 | 0 | 0 | 0 | 3 | 0 | 0 | 4 | 4 | 6 |
| | Mn 160 F.C. | 3 | 1 | 0 | 5 | 0 | 5 | 0 | 0 | 0 | 0 | 0 | 0 | 0 | 0 | 0 | 4 | 5 | 0 | 0 | 1 | 10 | 0 | 2 | 3 | 6 | 6 |
| | Mn 162 F.C. | 1 | 0 | 0 | 0 | 0 | 1 | 0 | 0 | 0 | 0 | 0 | 0 | 0 | 0 | 0 | 5 | 4 | 0 | 0 | 0 | 0 | 0 | 0 | 2 | 4 | 5 |
| | Mn 154 F.C. | 1 | 0 | 0 | 2 | 0 | 1 | 0 | 0 | 0 | 0 | 0 | 0 | 1 | 0 | 1 | 1 | 0 | 0 | 1 | 1 | 3 | 1 | 0 | 1 | 4 | 1 |
| | Mn 164 F.C. | 0 | 0 | 0 | 0 | 0 | 1 | 0 | 0 | 0 | 0 | 0 | 0 | 1 | 0 | 1 | 1 | 0 | 1 | 0 | 1 | 1 | 1 | 0 | 1 | 6 | 1 |
| | Mn 173 F.C. | 1 | 0 | 0 | 1 | 0 | 1 | 0 | 0 | 0 | 1 | 0 | 0 | 1 | 0 | 1 | 1 | 0 | 0 | 1 | 1 | 2 | 1 | 0 | 1 | 4 | 0 |
| | *O.* sp.[1] 127 O.C. | 5 | 0 | 0 | 5 | 3 | 6 | 0 | 0 | 0 | 1 | 0 | 0 | 0 | 0 | 0 | 6 | 8 | 0 | 0 | 3 | 0 | 0 | 3 | 5 | 16 | 6 |
| | *O.* sp. 128 O.C. | 6 | 0 | 0 | 4 | 2 | 6 | 0 | 0 | 0 | 2 | 0 | 0 | 0 | 0 | 0 | 4 | 6 | 0 | 0 | 3 | 2 | 0 | 3 | 3 | 27 | 5 |
| | *O.* sp. 159 O.C. | 4 | 0 | 0 | 3 | 2 | 5 | 0 | 0 | 0 | 1 | 0 | 0 | 0 | 0 | 0 | 5 | 7 | 0 | 0 | 3 | 1 | 0 | 3 | 3 | 18 | 6 |
| | *O.* cf. *badius* 145 O.C. | 2 | 0 | 0 | 1 | 4 | 5 | 0 | 0 | 0 | 0 | 0 | 0 | 0 | 0 | 0 | 4 | 6 | 0 | 0 | 3 | 0 | 0 | 4 | 3 | 24 | 3 |
| | *O.* cf. *badius* 150 O.C. | 2 | 0 | 0 | 4 | 4 | 7 | 0 | 0 | 0 | 0 | 0 | 0 | 0 | 0 | 0 | 4 | 6 | 0 | 0 | 3 | 0 | 2 | 5 | 3 | 15 | 5 |
| | *O.* cf. *badius* 151 O.C. | 2 | 0 | 0 | 3 | 4 | 4 | 0 | 0 | 0 | 0 | 0 | 0 | 0 | 0 | 0 | 5 | 9 | 0 | 0 | 4 | 0 | 0 | 4 | 3 | 23 | 3 |
| | Mn 156 O.C. | 8 | 0 | 0 | 4 | 2 | 5 | 0 | 1 | 0 | 1 | | 1 | 0 | 0 | 0 | 9 | 13 | 0 | 0 | 0 | 0 | 0 | 3 | 5 | 5 | 12 |
| | Mn 160 O.C. | 8 | 0 | 0 | 8 | 4 | 11 | 2 | 3 | 0 | 4 | 1 | 2 | 0 | 0 | 0 | 3 | 4 | 0 | 0 | 1 | 3 | 0 | 5 | 2 | 4 | 9 |
| | Mn 162 O.C. | 4 | 0 | 0 | 1 | 0 | 1 | 0 | 0 | 0 | 0 | 0 | 1 | 0 | 0 | 0 | 1 | 3 | 0 | 0 | 0 | 0 | 0 | 0 | 0 | 3 | 3 |
| | Mn 154 O.C. | 4 | 0 | 1 | 5 | 3 | 11 | 4 | 5 | 1 | 5 | 2 | 8 | 2 | 1 | 2 | 15 | 11 | 1 | 7 | 2 | 3 | 1 | 5 | 11 | 4 | 10 |
| | Mn 164 O.C. | 3 | 0 | 0 | 2 | 1 | 2 | 0 | 1 | 1 | 1 | 0 | 1 | 1 | 1 | 1 | 2 | 0 | 1 | 1 | 2 | 1 | 1 | 1 | 1 | 5 | 2 |
| | Mn 173 O.C. | 2 | 0 | 0 | 3 | 1 | 2 | 0 | 0 | 1 | 1 | 0 | 1 | 1 | 1 | 1 | 1 | 0 | 1 | 1 | 2 | 2 | 1 | 1 | 1 | 4 | 1 |
| | *O.* sp. 127 (2016) O. mj. w. guts | 1 | 0 | 0 | 2 | 0 | 1 | 0 | 0 | 1 | 1 | 0 | 0 | 1 | 0 | 1 | 1 | 0 | 0 | 1 | 2 | 1 | 1 | 0 | 1 | 2 | 1 |
| | Mn 154 O. mj. w. guts | 0 | 0 | 0 | 3 | 1 | 1 | 0 | 0 | 1 | 1 | 0 | 0 | 1 | 1 | 1 | 1 | 0 | 1 | 1 | 2 | 1 | 1 | 1 | 1 | 1 | 0 |
| | Mn 164 O. mj. w. guts | 0 | 0 | 0 | 1 | 1 | 1 | 0 | 0 | 1 | 1 | 0 | 1 | 1 | 1 | 1 | 1 | 0 | 1 | 2 | 2 | 1 | 1 | 0 | 1 | 2 | 1 |
| | Mn 173 O. mj. w. guts | 0 | 0 | 0 | 1 | 0 | 2 | 0 | 0 | 1 | 1 | 0 | 0 | 1 | 0 | 1 | 1 | 0 | 1 | 1 | 2 | 1 | 1 | 0 | 1 | 1 | 0 |
| NaOH | *O.* sp.[1] 127 F.C. (2015-2016) | 21 | 48 | 31 | 36 | 3 | 13 | 10 | 16 | 0 | 2 | 38 | 45 | 3 | 18 | 0 | 10 | 32 | 0 | 30 | 6 | 4 | 5 | 35 | 3 | 16 | 0 |
| | *O.* sp. 128 F.C. | 21 | 48 | 40 | 37 | 3 | 13 | 12 | 17 | 0 | 2 | 36 | 44 | 3 | 18 | 0 | 9 | 29 | 0 | 42 | 9 | 4 | 6 | 35 | 2 | 14 | 0 |
| | *O.* sp. 159 F.C. | 21 | 50 | 20 | 28 | 5 | 16 | 40 | 27 | 0 | 2 | 32 | 41 | 5 | 27 | 0 | 9 | 26 | 0 | 17 | 3 | 3 | 7 | 39 | 0 | 9 | 0 |
| | *O.* cf. *badius* 145 F.C. | 19 | 42 | 13 | 38 | 4 | 10 | 8 | 17 | 0 | 1 | 30 | 36 | 3 | 19 | 0 | 5 | 21 | 0 | 12 | 2 | 2 | 5 | 35 | 0 | 8 | 0 |
| | *O.* cf. *badius* 150 F.C. | 20 | 49 | 18 | 30 | 4 | 17 | 9 | 17 | 0 | 1 | 33 | 41 | 4 | 26 | 0 | 4 | 17 | 0 | 14 | 3 | 3 | 6 | 35 | 0 | 11 | 0 |
| | *O.* cf. *badius* 151 F.C. | 21 | 50 | 18 | 29 | 7 | 16 | 28 | 27 | 0 | 2 | 34 | 43 | 6 | 31 | 0 | 9 | 29 | 0 | 15 | 3 | 3 | 9 | 38 | 1 | 10 | 0 |
| | Mn 156 F.C. | 22 | 53 | 5 | 26 | 3 | 12 | 12 | 19 | 0 | 2 | 38 | 52 | 4 | 17 | 0 | 8 | 26 | 0 | 4 | 3 | 4 | 3 | 36 | 4 | 9 | 0 |
| | Mn 160 F.C. | 21 | 46 | 20 | 30 | 4 | 13 | 14 | 21 | 0 | 3 | 37 | 47 | 3 | 18 | 0 | 6 | 24 | 0 | 18 | 3 | 9 | 4 | 37 | 7 | 12 | 0 |
| | Mn 162 F.C. | 22 | 55 | 2 | 25 | 2 | 12 | 12 | 19 | 0 | 2 | 38 | 55 | 4 | 19 | 0 | 8 | 26 | 0 | 1 | 3 | 2 | 4 | 35 | 3 | 6 | 0 |
| | Mn 154 F.C. | 9 | 56 | 16 | 27 | 2 | 17 | 12 | 19 | 2 | 5 | 49 | 81 | 5 | 17 | 4 | 2 | 2 | 1 | 23 | 6 | 11 | 6 | 29 | 1 | 40 | 1 |
| | Mn 164 F.C. | 12 | 62 | 3 | 31 | 1 | 13 | 12 | 21 | 2 | 3 | 50 | 90 | 7 | 14 | 5 | 1 | 0 | 1 | 3 | 5 | 7 | 4 | 28 | 0 | 33 | 1 |
| | Mn 173 F.C. | 13 | 61 | 9 | 27 | 5 | 14 | 16 | 20 | 2 | 3 | 45 | 79 | 4 | 19 | 5 | 3 | 2 | 1 | 11 | 7 | 13 | 6 | 32 | 0 | 29 | 0 |
| | *O.* sp.[1] 127 O.C. (2015-2016) | 20 | 34 | 25 | 42 | 3 | 11 | 8 | 13 | 0 | 0 | 28 | 35 | 0 | 11 | 0 | 4 | 19 | 0 | 24 | 2 | 3 | 4 | 33 | 0 | 10 | 0 |
| | *O.* sp. 128 O.C. | 21 | 46 | 26 | 35 | 4 | 14 | 34 | 23 | 0 | 2 | 32 | 39 | 3 | 20 | 0 | 8 | 23 | 0 | 25 | 3 | 3 | 6 | 36 | 1 | 10 | 0 |

| Treatment | Sample | (1->3) β-D-glucan (BS-400-2) | (1->3)(1->4) β-D-glucan (BS-400-3) | (1->4) β-D-mannan (BS-400-4) | Cellulose (CBM3a) | Extensin (LM1) | Arabinogalactan protein AGP (LM2) | (1->4) β-D-galactan (LM5) | (1->5) α-L-arabinan (DLM6) | Homogalacturonan with an intermediate DE (LM7) | Xylogalacturonan (LM8) | (1->4) β-D-xylan (LM10) | Xylan/Arabinoxylan (LM11) | Arabinan (LM13) | Xyloglucan (LM15) | Arabinan (LM16) | Homogalacturonan (LM18 (MUC2)) | Homogalacturonan (LM19 (XGA2)) | Homogalacturonan (LM20 (MUC1)) | Mannan (LM21) | Mannan LM22 | (1->4) β-D-xylan (LM23) | Xyloglucan (LM24) | Xyloglucan (LM25) | Homogalacturonan, low DE (JIM5) | No defined epitope, (Anti callose, MLG like binding) (JIM6) | Homogalacturonan, low DE (JIM7) |
|---|---|---|---|---|---|---|---|---|---|---|---|---|---|---|---|---|---|---|---|---|---|---|---|---|---|---|---|
| | *O.* sp. 159 O.C. | 10 | 33 | 19 | 34 | 4 | 10 | 5 | 13 | 2 | 3 | 33 | 55 | 3 | 13 | 4 | 2 | 2 | 1 | 31 | 5 | 12 | 6 | 29 | 1 | 24 | 0 |
| | *O.* cf. *badius* 145 O.C. | 11 | 29 | 4 | 42 | 2 | 2 | 4 | 7 | 0 | 0 | 23 | 29 | 0 | 4 | 0 | 2 | 10 | 0 | 3 | 0 | 0 | 2 | 23 | 0 | 6 | 0 |
| | *O.* cf. *badius* 150 O.C. | 15 | 42 | 5 | 37 | 3 | 4 | 9 | 17 | 0 | 0 | 29 | 36 | 2 | 9 | 0 | 8 | 26 | 0 | 4 | 0 | 0 | 3 | 31 | 0 | 9 | 0 |
| | *O.* cf. *badius* 151 O.C. | 15 | 45 | 5 | 34 | 3 | 7 | 40 | 19 | 0 | 0 | 23 | 35 | 3 | 12 | 0 | 6 | 18 | 0 | 3 | 0 | 0 | 3 | 30 | 0 | 8 | 0 |
| | Mn 156 O.C. | 23 | 53 | 7 | 39 | 2 | 13 | 16 | 23 | 0 | 2 | 42 | 50 | 3 | 17 | 0 | 4 | 19 | 0 | 6 | 2 | 3 | 4 | 36 | 2 | 12 | 0 |
| | Mn 160 O.C. | 20 | 39 | 20 | 35 | 6 | 14 | 18 | 27 | 0 | 2 | 35 | 43 | 3 | 17 | 0 | 11 | 32 | 0 | 20 | 2 | 4 | 4 | 38 | 3 | 10 | 0 |
| | Mn 162 O.C. | 24 | 54 | 8 | 36 | 3 | 15 | 17 | 26 | 0 | 2 | 43 | 52 | 4 | 19 | 0 | 6 | 23 | 0 | 6 | 2 | 3 | 4 | 37 | 1 | 12 | 0 |
| | Mn 154 O.C. | 10 | 28 | 19 | 31 | 2 | 13 | 10 | 16 | 1 | 3 | 33 | 49 | 2 | 13 | 3 | 2 | 1 | 1 | 33 | 6 | 10 | 6 | 29 | 0 | 31 | 0 |
| | Mn 164 O.C. | 9 | 38 | 4 | 27 | 1 | 11 | 8 | 18 | 1 | 3 | 29 | 44 | 2 | 12 | 3 | 1 | 0 | 1 | 5 | 1 | 7 | 4 | 24 | 0 | 39 | 1 |
| | Mn 173 O.C. | 13 | 47 | 5 | 26 | 4 | 12 | 11 | 17 | 2 | 2 | 34 | 61 | 3 | 15 | 5 | 2 | 1 | 1 | 8 | 4 | 8 | 5 | 24 | 0 | 29 | 0 |
| | *O.* sp. 127 O. mj. w. guts | 3 | 9 | 3 | 9 | 1 | 2 | 0 | 1 | 2 | 1 | 10 | 18 | 1 | 5 | 3 | 1 | 0 | 0 | 5 | 3 | 3 | 2 | 12 | 0 | 5 | 1 |
| | Mn 154 O. mj. w. guts | 4 | 12 | 1 | 8 | 1 | 3 | 1 | 3 | 1 | 1 | 4 | 12 | 1 | 2 | 2 | 2 | 0 | 1 | 1 | 3 | 3 | 1 | 8 | 0 | 0 | 0 |
| | Mn 164 O. mj. w. guts | 5 | 24 | 1 | 17 | 1 | 4 | 2 | 6 | 2 | 2 | 18 | 34 | 2 | 6 | 3 | 1 | 0 | 0 | 1 | 3 | 3 | 2 | 14 | 0 | 0 | 1 |
| | Mn 173 O. mj. w. guts | 7 | 15 | 1 | 11 | 1 | 3 | 2 | 4 | 2 | 1 | 8 | 18 | 2 | 5 | 3 | 1 | 0 | 0 | 1 | 4 | 3 | 2 | 13 | 0 | 5 | 1 |

Table S11: Comprehensive microarray polymer profiling heatmap with cut off of 5 imposed to remove background signal. The numbers represent the intensity of the signal. White represents the lowest, yellow and orange intermediate, and red highest signals. *O.* sp: *Odontotermes* sp., *O.* cf. *badius*: *Odontotermes* cf. *badius*, Mn: *Macrotermes natalensis*, F.C.: Fresh comb, O.C.: Old comb, O. mj. w. guts: Old major worker guts. [1]This colony was sampled in 2015 and 2016, but there was no significant effect of year (unpaired t-test; $t_{24} = 1.710$, p = 0.1453), so we report averages of the measurements.

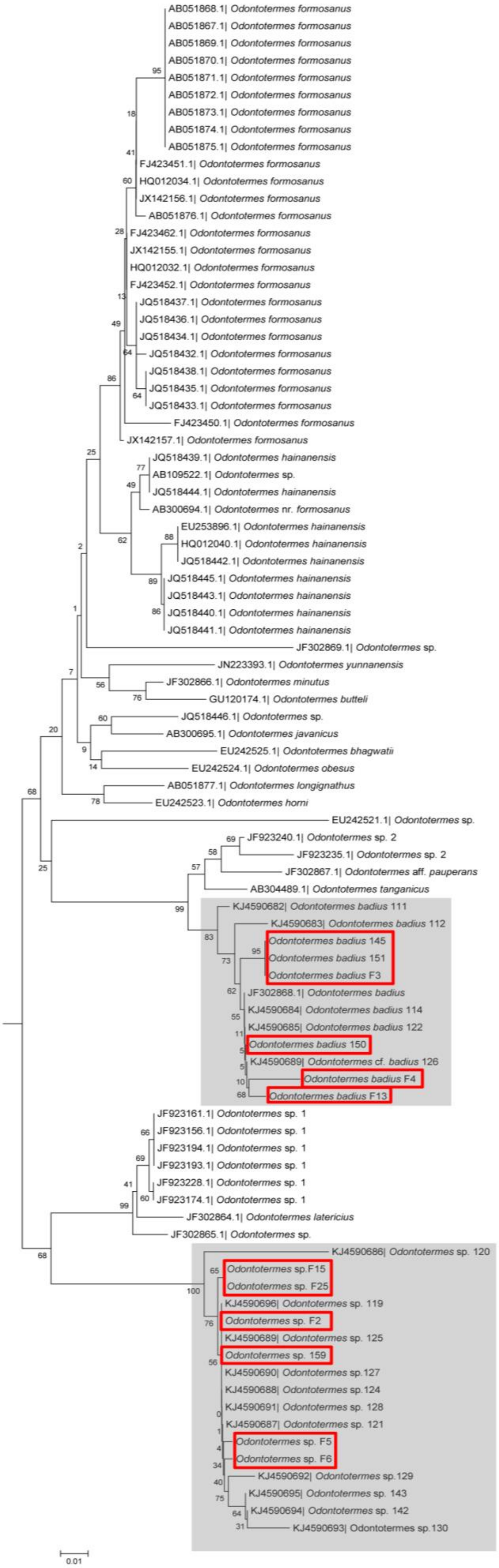

Figure S1: Phylogenetic analysis placing the *Odontotermes* COII sequences from foraging sites and nests used for the enzyme, polymer content, and RNAseq in a phylogeny with *Odontotermes* sequences from Otani et al. (2014) and GenBank. Six of our samples were *O.* cf. *badius*, while the remaining samples group within a well-supported clade named *Odontotermes* sp. by as Otani et al. (2014). Bootstrap support based on 10,000 pseudo-replicates under Neighbor-Joining conditions

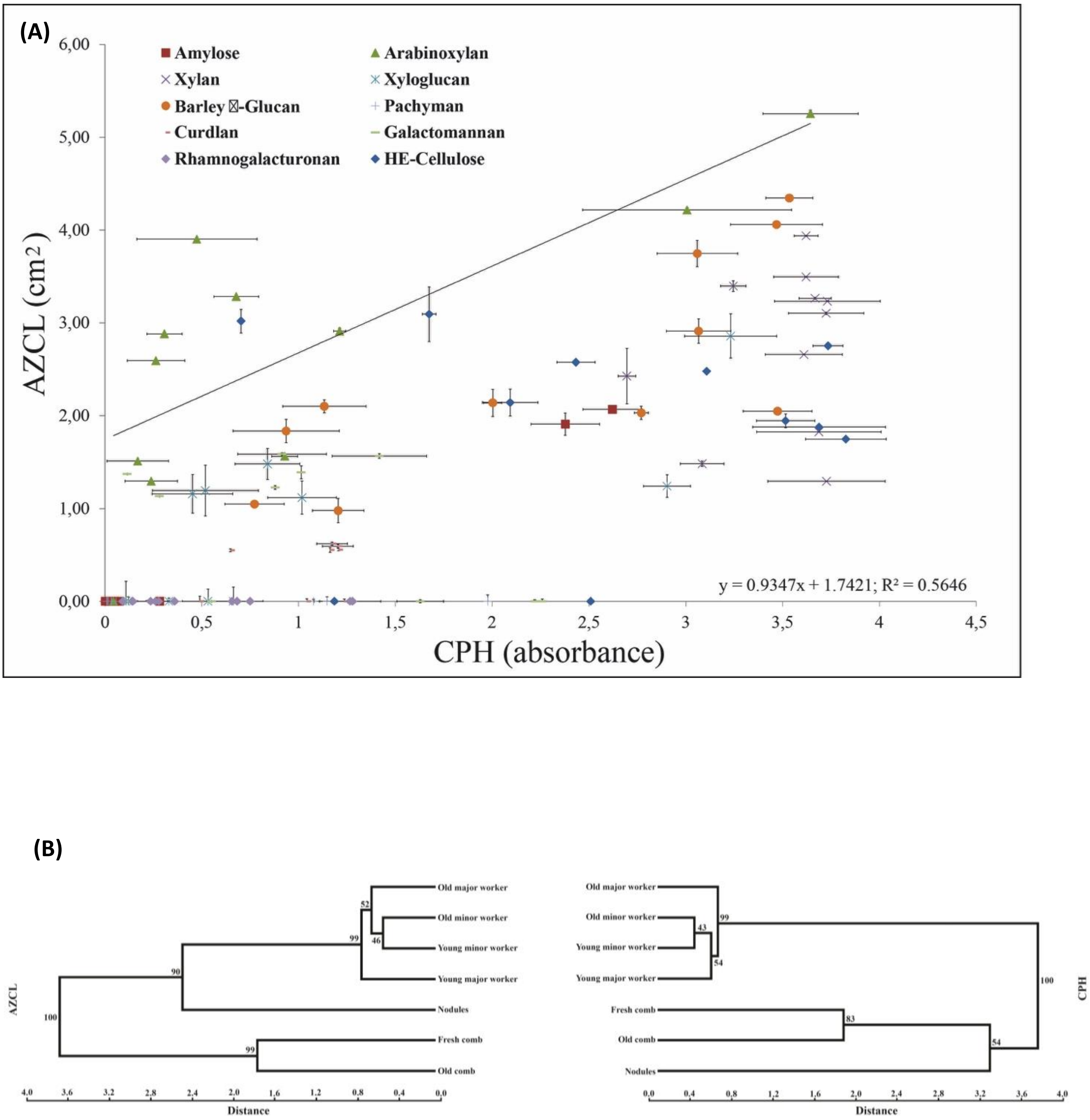


Figure S2: Comparison of CPH and AZCL substrates enzyme screening**. (a)** Scatter plot showing the relationship between CPH (x-axis) and AZCL (y-axis) substrate enzyme activities (mean ± SE; n=3). **(b)** Euclidean distance clustering analysis with bootstrap support after 10,000 permutations showing that the techniques provide somewhat comparable results.

**Supplemental references**

Clausen MH, Willats WGT, Knox JP. 2003. Synthetic methyl hexagalacturonatehapten inhibitors of anti homogalacturonan monoclonal antibodies LM7, JIM5 and JIM7. Carbohydr Res 17: 1797–1800.

Jones L, Seymour CB, Knox JP. 1997. Localization of pectic galactan in tomato cell walls using a monoclonal antibody specific to (1→4)-β-D-galactan. Plant Physiol 4: 1405–1412.

Kračun SK, Schückel J, Westereng B, Thygesen LG, Monrad RN, Eijsink V, Willats WGT .2015. A new generation of versatile chromogenic substrates for high-throughput analysis of biomass-degrading enzymes. Biotechnol Biofuels 8: 70.

Marcus SE, Blake AW, Benians TAS, Lee KJD, Poyser C, Donaldson L, Leroux O, Rogowski A, Petersen HL, Boraston A, Gilbert HJ, Willats WGT, Knox JP. 2010. Restricted access of proteins to mannan polysaccharides in intact plant cell walls. Plant J 64: 191–203.

Marcus SE, Verhertbruggen Y, Hervé C, Ordaz-Ortiz JJ, Farkas V, Pedersen HL, Willats WGT, Knox JP. 2008. Pectic homogalacturonan masks abundant sets of xyloglucan epitopes in plant cell walls. BCM Plant Biol 8: 60.

McCartney L, Marcus SE, Knox JP. 2005. Monoclonal antibodies to plant cell wall xylans and arabinoxylans. J Histochem Cytochem 4: 543–546.

Meikle PJ, Hoogenraad NJ, Bonig I, Clarke AE, Stone BA. 1994. A (1→3,1→4)-β-glucan-specific monoclonal antibody and its use in the quantitation and immunocytochemical location of (1→,1→4)-β-glucans. Plant J 5: 1–9.

Meikle PJ, Bonig I, Hoogenraad NJ, Clarke AE, Stone BA. 1991. The location of (1→ 3)-β-glucans in the walls of pollen tubes of *Nicotianaalata* using α-(1 3)-β-glucan-specific monoclonal antibody. Planta 1:1–8.

Moller I, Marcus SE, Harger A, Verhertbruggen Y, Verhoef R, Schols H, Ulvskov P, Mikkelsen JD, Knox JP, Willats WGT. 2008. High-throughput screening of monoclonal antibodies against plant cell wall glycans by hierarchical clustering of their carbohydrate microarray binding profiles. Glycoconj J 25: 37–48.

Otani S, Mikaelyan A, Nobre T, Hansen LH, Koné NA, Sørensen SJ, Aanen DK, Boomsma JJ, Brune A, Poulsen M. 2014. Identifying the core microbial community in the gut of fungus-growing termites. Mol Ecol 23: 4631-4644

Pedersen HL, Fangel JU, McCleary B, Ruzanski C, Rydahl MG., Ralet MC, Farkas V, von Schantz L, Marcus SE, Andersen MC, Field R, Ohlin M, Knox JP, Clausen MH, Willats WGT. 2012. Versatile high resolution oligosaccharide microarrays for plant glycobiology and cell wall research. J Biol Chem 47: 39429–39438.

Pettolino FA, Hoogenraad NJ, Ferguson C, Prof TB, Johnson E, Stone BA. 2001. A (1→4)-β-mannan-specific monoclonal antibody and its use in the immunocytochemical location of galactomannans. Planta 2: 235–242.

Smallwood M, Martin H, Knox JP. 1995. An epitope of rice threonine- and hydroxyproline-rich glycoprotein is common to cell wall and hydrophobic plasma-membrane glycoproteins. Planta 3: 510–522.

Tormo J, Jamed R, Chirinol AJ, Morag E, Bayer EA, Shoham Y, Steitz TA. 1996. Crystal structure of a bacterial family-III cellulose-binding domain: a general mechanism for attachment to cellulose. EMBO J 15: 5739–5751.

Verhertbruggen Y, Marcus SE, Harger A, Verhoef R, Schols H, McCleary BV, McKee L, Golbert HJ, Knox JP. 2009. Developmental complexity of arabinan polysaccharides and their processing in plant cell walls. Plant J 59: 413–425.

Willats GTW, Marcus SE, Knox JP. 1998. Generation of a monoclonal antibody specific to (1→5)-α-L-arabinan. Carbohydr Res 6: 131–139.

Willats GTW, McCartney L, Steele-King CG, Marcus SE, Mort A, Huismanm M, Van Alebeek GJ, Schols HA, Voragen AG, Le Goff A, Bonnin E, Thibault JF, Knox JP. 2004. A xylogalacturonan epitope is specifically associated with plant cell detachment. Planta. **4**:673–681.

Willats GTW, Orfila C, Limberg G, Buchholt HC, Alebeek GJWMV, Voragen AGJ, Christensen TM, Mikkelsen JD, Murray BS, Knox JP. 2001. Modulation of the degree and pattern of methyl-sterification of pectic homogalacturonan in plant cell walls. J Biol Chem 22: 19404–19413.

Yates EA, Valdor JF, Haslam SM, Morris HR, Dell A, Mackie W, Knox JP. 1996. Characterization of carbohydrate structural features recognized by anti-arabinogalactan-protein monoclonal antibodies. Glycobiology 2: 131–139.
